# Evidence for Population-Specific Positive Selection on Immune Genes of *Anopheles gambiae*

**DOI:** 10.1534/g3.112.004473

**Published:** 2012-12-01

**Authors:** Jacob E. Crawford, Emmanuel Bischoff, Thierry Garnier, Awa Gneme, Karin Eiglmeier, Inge Holm, Michelle M. Riehle, Wamdaogo M. Guelbeogo, N’Fale Sagnon, Brian P. Lazzaro, Kenneth D. Vernick

**Affiliations:** * Department of Entomology, Cornell University, Ithaca, New York 14853; † Unit of Insect Vector Genetics and Genomics, Department of Parasitology and Mycology, Institut Pasteur, F-75015 Paris, France; ‡ Centre National de la Recherche Scientifique (CNRS), URA 3012, F-75015 Paris, France; § Centre National de Recherche et de Formation sur le Paludisme, 01 BP 2208 Ouagadougou, Burkina Faso, and; ** Microbial and Plant Genomics Institute, Department of Microbiology, University of Minnesota, Saint Paul, Minnesota 55108

**Keywords:** *Anopheles gambiae*, positive selection, host−pathogen interaction, immune gene, *Plasmodium falciparum*

## Abstract

Host-pathogen interactions can be powerful drivers of adaptive evolution, shaping the patterns of molecular variation at the genes involved. In this study, we sequenced alleles from 28 immune-related loci in wild samples of multiple genetic subpopulations of the African malaria mosquito *Anopheles gambiae*, obtaining unprecedented sample sizes and providing the first opportunity to contrast patterns of molecular evolution at immune-related loci in the recently discovered GOUNDRY population to those of the indoor-resting M and S molecular forms. In contrast to previous studies that focused on immune genes identified in laboratory studies, we centered our analysis on genes that fall within a quantitative trait locus associated with resistance to *Plasmodium falciparum* in natural populations of *A. gambiae*. Analyses of haplotypic and genetic diversity at these 28 loci revealed striking differences among populations in levels of genetic diversity and allele frequencies in coding sequence. Putative signals of positive selection were identified at 11 loci, but only one was shared by two subgroups of *A. gambiae*. Striking patterns of linkage disequilibrium were observed at several loci. We discuss these results with respect to ecological differences among these strata as well as potential implications for disease transmission.

More than six decades ago, J. B. S. Haldane inferred from simple ecological observations that host-pathogen interactions must be a unique and powerful driving agent of adaptive evolution (Haldane 1949). Such evolutionary dynamics are expected to leave traces in genomes of both host and pathogen, especially in immunity-related genes for the former and in virulence genes for the latter. Consistent with this expectation, molecular evolutionary studies of genes in primates, Drosophila, and plants have shown that immune-related genes tend to evolve adaptively and are among the most rapidly evolving genes in the genome (Clark *et al.* 2003; Schlenke and Begun 2003; Nielsen *et al.* 2005; Tiffin and Moeller 2006; Sackton *et al.* 2007). Here, we examine patterns of genetic variation in multiple natural populations of the African malaria mosquito, *Anopheles gambiae s.s*., to identify possible evidence of pathogen-driven molecular evolution.

One form of pathogen-driven evolution is positive selection acting on a beneficial allele or alleles. In the classical selective sweep model, positive selection acts on a new allele that arises by mutation, driving it to fixation within a population or species (Maynard Smith and Haigh 1974). Partial selective sweeps are also possible, in which the beneficial mutation increases in frequency within the population but does not or has not yet reached fixation (Hudson *et al.* 1997). Positive selection also can act on polymorphisms segregating at low or intermediate frequency in the population before the selective event. Such selection on standing genetic variation can yield a rapid response to a change in selective pressure, such as a switch of ecological niche (Przeworski *et al.* 2005; Pritchard *et al.* 2010).

Identifying adaptive evolution in *A. gambiae s.s*. also may have implications for human health because this species is a primary vector of the human malaria parasite *Plasmodium falciparum* in sub-Saharan Africa. In numerous studies authors have examined patterns of nucleotide substitution in an effort to find signatures of positive selection in immune genes of *A. gambiae*, its subgroups and its sister taxon *Anopheles arabiensis*. Thus far, there is little reported evidence for strong directional or balancing selection, with some genes showing strong purifying selection (Obbard *et al.* 2007, 2008; Slotman *et al.* 2007; Cohuet *et al.* 2008). These studies also have highlighted the challenge of using existing tests of selection in a species with the phylogenetic history of *A. gambiae* (Obbard *et al.* 2007). Two recent studies reported parallel selective sweeps among M-form *A. gambiae* in two genes whose protein products have been shown to interact (Rottschaefer *et al.* 2011; White *et al.* 2011). Here we use similar approaches to examine patterns of nucleotide variation in population subgroups of a deeply sampled local population from Burkina Faso, West Africa.

Natural mosquito phenotypic variation for susceptibility to malaria parasite infection has a large genetic component, and has been mapped to loci on all three chromosomes in multiple studies in both West and East Africa (Niaré *et al.* 2002; Menge *et al.* 2006; Riehle *et al.* 2006, 2007). In particular, the left arm of chromosome 2 carries a genomic region containing quantitative trait loci that has been termed a “Plasmodium-Resistance Island” (Menge *et al.* 2006; Riehle *et al.* 2006; 2007). The PRI is ~15 megabases (Mb) in size and contains ~1000 coding genes (Riehle *et al.* 2006); however, the causative genetic variant(s) underlying the mapped quantitative trait loci have not been identified. Here we analyzed sequence diversity in a large sample of wild-caught mosquitoes at a subset of the PRI candidate genes to analyze the selective history and genetic structure at these loci.

*Anopheles gambiae s.s*. comprises genetically differentiated but morphologically identical subgroups that can be distinguished only on the basis of molecular diagnostic assays, thus termed “molecular forms.” The “S” molecular form has the largest range and is widespread throughout sub-Saharan Africa. The “M” molecular form has arisen only in West Africa (della Torre *et al.* 2001), likely as a population derived from the S form, and the two are broadly sympatric over the M-form range. Over much of their range, M and S forms are reproductively isolated at the prezygotic level (Diabaté *et al.* 2007) and display restricted gene flow (Wondji *et al.* 2005; Neafsey *et al.* 2010). However, recent work suggests that the evolutionary status of the subgroups may be more dynamic than previously thought because some sympatric populations of M and S forms have been shown to have increased levels of hybridization, with complex patterns of directional introgression (Oliveira *et al.* 2008; Marsden *et al.* 2011).

We identified an additional population form in the Sudan Savanna zone of Burkina Faso, the GOUNDRY subgroup, which is genetically distinct from both M and S forms, and in which the markers typically diagnostic for the M and S forms segregate freely (Riehle *et al.* 2011). In this sampling location, the three subgroups are sympatric and larval collections yield all three forms. ENDO M and ENDO S forms (hereafter referred to as M and S molecular forms) can differ by the ecotype of their larval sites, where the S form may favor temporary breeding sites and the M form may prefer permanent freshwater pools (Costantini *et al.* 2009), although at the current study site in Burkina Faso and elsewhere, M and S larval breeding sites also often are shared. The larval ecology of GOUNDRY is not well understood, but in the study zone GOUNDRY larvae appear to inhabit different types of larval sites as compared to sites of the M and S forms combined (M. M. Riehle, K. D. Vernick, N. Sagnon, and W. M. Guelbeogo, unpublished observation).

The larval habitats of these mosquitoes harbor a diverse community of invertebrates, microbes, fungi, and protozoa, and there is some evidence that the M and S molecular forms may differ in their interactions with this community, particularly with respect to their ability to avoid predators that are more often found in habitats preferred by the M-form population (Diabaté *et al.* 2008; Fillinger *et al.* 2009; Gimonneau *et al.* 2010; 2012). At the adult stage, the M and S molecular forms are highly endophilic, tending to rest indoors after feeding, and are thus referred to collectively as ENDO forms (Riehle *et al.* 2011). In contrast, GOUNDRY adults presumably exploit yet undiscovered outdoor resting sites, although occasional indoor-resting GOUNDRY adults have been captured (M. M. Riehle, K. D. Vernick, N. Sagnon, and W. M. Guelbeogo, unpublished observation). Our previous report shows GOUNDRY to be inherently more susceptible to *P. falciparum* infections than either M or S form; however, its role as a natural vector of malaria remains an open question and an active area of research (Riehle *et al.* 2011).

The genomic interval containing the Plasmodium-Resistance Island partially overlaps the large 2La paracentric chromosomal inversion. 2La polymorphism is widespread in Africa and originally was noted for the allele frequency correlation with degree of environmental aridity (Coluzzi *et al.* 1979; Powell *et al.* 1999). In the Burkina Faso study area, the M and S form populations are nearly fixed for the inverted 2La^a^ arrangement, whereas the GOUNDRY subgroup segregates both the standard 2La^+^ and inverted 2La^a^ arrangements in Hardy-Weinberg equilibrium (Riehle *et al.* 2011).

In this study, we used a population genetic approach to test hypotheses of adaptive evolution at candidate immune genes within the PRI infection control locus. The candidate gene set consists of genes with a diverse array of putative immune functions, including immune effector molecules, pathogen recognition, signal transduction, and modulation (Riehle *et al.* 2006). We hypothesized that the differences in ecology and adaptation of the M and S molecular forms, the 2La inversion arrangements, and the GOUNDRY subgroup might result in exposure to distinct suites of pathogens in the environment and that these differences in exposure could result in different host−pathogen evolutionary dynamics that may have important implications for malaria transmission. We analyzed the patterns of genetic variation in 28 immunity-related genes in four population strata of *A. gambiae* collected from the village of Goundry, Burkina Faso. We find that signals of putative positive selection vary among the genetic subpopulations and 2La inversion types. We discuss these results in the context of Plasmodium selection pressure and transmission, ecological differences among these populations, and the ongoing incipient speciation process.

## Materials and Methods

### Mosquito collection and sample sets

The mosquitoes used in this study were collected as described by Riehle *et al.* (2011), with full details on the origin of mosquito specimen, sample composition, and genotyping methodologies given in that publication. In summary, *A. gambiae s.s*. were collected as larvae in 2007 and 2008 from larval habitats in and around Goundry, Burkina Faso (coordinates 12°30′N, 1°20′W), about 30 km north of the capital city Ouagadougou. The collection area is situated within the Sudan-Savanna (Sudano-Sahelian) ecological zone of tropical shrubland and dry forest. Mosquitoes from 56 larval collections were reared to adults in an insectary. Sixteen distinct larval collections from the years 2007 and 2008 were used to compose the samples.

### DNA isolation

DNA was extracted from individual adult female mosquito carcasses in 100 µL of DNAzol (Invitrogen) according to the manufacturer’s recommendations. The genomic DNA from each mosquito was resuspended in 500 µL of H_2_O.

### Genotyping and population assignment

Species, molecular form, and 2La inversion karyotype were determined as described (Riehle *et al.* 2011). In brief, species and molecular form were typed using the SINE200 X6.1 assay (Santolamazza *et al.* 2008). The 2La inversion was typed using a published molecular assay (White *et al.* 2007). Fluorescent primers were used in both assays, and polymerase chain reaction (PCR) fragments were sized using an ABI Genetic Analyzer 3730 as previously described (Riehle *et al.* 2011).

Genotyping of microsatellites on the third chromosome was carried out as described previously (Riehle *et al.* 2011). The genotyped markers were: 3R.H59, 3R.H93, 3R.H249, 3R.H119, 3R.H555, 3L.H242, 3L.H758, and 3L.H817. These markers are regularly spaced on the two chromosome arms, present no detectable null alleles, and segregate at HWE. Microsatellites were used to assign the samples to either ENDO (M and S) or GOUNDRY subpopulations using the program STRUCTURE (Pritchard *et al.* 2000), and the standard molecular diagnostic (Favia *et al.* 2001) was used to assign M *vs.* S molecular form as described in Riehle *et al.* 2011. Mosquitoes not assigned to a single cluster with greater than 80% probability by STRUCTURE were removed from the analysis (~1.9%, eight mosquitoes). These few ambiguous assignments were due to missing genotype data. Heterokaryotypic 2La^a^/2La^+^ mosquitoes were not included in this study. Mosquito identifiers, sampling year, molecular form status, and 2La karyotype for each mosquito are provided in Supporting Information, Table S1.

### PCR amplification of target gene fragments

For 28 selected candidate genes, two alternative primer pairs were designed manually on the basis of the *A. gambiae* genome (Vectorbase, *A. gambiae* genome, version AgamP3). Primers were designed in coding exons to generate a PCR product of ~500 bp. If possible, the PCR amplicon was designed to span an intron to increase the number of sequence variants. The two alternative primer pairs were first tested for efficient amplification with DNAs from three unrelated *A. gambiae* s.s. of different 2L inversion karyotypes (2La^a^/2La^a^, 2La^+^/2La^+^, 2La^+^/2La^a^) and one mosquito from the Ngousso colony of *A. gambiae*. The generated PCR amplicons were analyzed on a 1% agarose gel, and single-band PCR products of the correct size were sequenced in both directions to confirm the amplification of the correct target gene. If needed, additional primer pairs were designed to optimize the amplification. The primer pair that performed best with all four mosquito DNAs was retained for the experiment with the field-collected specimens. The sequences of the primers are given in Table S2. In the sample set from 2007, PCR fragments were generated from all 28 target genes. In the 2008 samples set PCR fragments were produced from 7 of the 28 target genes (Table S1). To facilitate the direct sequencing of the PCR products, all retained primer pairs were synthesized with 5′ extensions corresponding to the universal forward and reverse M13 primers, respectively.

PCR reactions were performed in 20 µL with 2 µL of genomic DNA using AccuPrime SuperMixII (Invitrogen) according to the supplier’s recommendations. The amplification conditions included an initial denaturation step of 94° for 3 min, followed by 40 cycles at 94° for 30 sec, 55° for 45 sec and 72° for 1 min, and a final extension step at 72° for 10 min. Unincorporated primer molecules and nucleotides were removed from the PCR product by centrifugation over Sephadex P100 columns in Multi Screen filter plates (Millipore).

### Sequencing

Sequencing reactions were conducted using 2 to 4 µL of the purified PCR product. All amplicons were sequenced in both directions using the universal forward and reverse M13 primers, the ABI Big Dye Terminator v.3.1 Cycle Sequencing kit (LifeTechnologies), and an ABI Prism 3730 DNA Analyzer (Applied Biosystems). Genotypes were called for each heterozygous sequence individually automatically using the internal software of the DNA Analyzer based on intensity ratios of the sequence chromatograms. We conducted an initial visual verification of sequence traces to confirm that the automated calling was accurate. The sequences were assembled using CodonCode Aligner (CodonCode Corporation). To assemble a contig, the two sequences of a PCR fragment from a single mosquito were aligned using the reference genome of *A. gambiae* as a guide. Then a multiple sequence alignment of all consensus sequences was constructed using ClustalW. Gene names, genomic locations, and AGAP identifiers are provided in Table S2 and Table S3. The number of chromosomes sequenced from each population for each fragment is provided in Table S4 and in the *Results* section. Sequence data from this article have been deposited with the EMBL/GenBank Data Libraries under accession nos. JX650224 to JX656698).

### Haplotype phasing

Haplotypes were inferred from the aligned sequences using PHASE 2.1.1 (Stephens *et al.* 2001) for each population independently using default options. The FASTA sequence alignment obtained for each population was converted into the input file format requested for phase inference using the program Seqphase (Flot 2010). The same software was also used to transform the PHASE output file back into FASTA. Within the GOUNDRY subgroup, for sequenced amplicons located within the 2La inversion, the phase reconstruction was done independently for 2La^a^/2La^a^ and 2La^+^/2La^+^ mosquitoes.

### Sequences of related taxa

For inference of ancestral *vs.* derived allele states and for reference within neighbor-joining gene trees, we used sequence from *Anopheles arabiensis*, *Anopheles quadriannulatus*, and *Anopheles merus*, all of which are members of the *A. gambiae* species complex. The phylogenetic relationships between these taxa can be problematic for use in population genetic and molecular evolutionary analysis because alleles from these species are not always reciprocally monophyletic (Obbard *et al.* 2007, 2009). *A. merus*, however, is the most diverged of these species and is therefore the best available for use in population genetic tests with *A. gambiae*. In the present study, *A. merus* is used as the outgroup for population genetic tests whereas the other taxa are only included for visual orientation in the neighbor-joining trees. To collect sequence for each gene from these species, we downloaded paired-end lanes of Illumina short read sequence data from the NCBI Short Read Archive (*A. merus*: SRR314654 and SRR314646; *A. arabiensis*: SRR314650; *A. quadriannulatus*: SRR314661), deposited by a public sequencing initiative (Besansky and Anopheles Genomes Cluster Committee 2008). These short reads were generated from whole-genome sequencing of a pool of two individuals from the *A. merus* OPHANSI strain, a pool of two individuals from the DONGOLA strain of *A. arabiensis*, and a pool of two individuals from the SKUQUA strain of *A. quadriannulatus*. Each paired-end lane was mapped to the *Anopheles gambiae* PEST genome sequence (AgamP3, August 2011 release from VectorBase.org) using BWA (Li and Durbin 2009) with default parameter settings except for the edit distance, which was set to 8 to accommodate the relatively high expected genetic distance between the reads and the reference. Read mapping resulted in median alignment depths of 20, 20, and 23 for *A. merus*, *A. arabiensis*, and *A. quadriannulatus*, respectively. We used the mpileup function in SAMtools (Li *et al.* 2009) to generate pileups and call variants. We extracted sequence for each species by substituting the alternative nucleotide into the *A. gambiae* reference sequence whenever the short-read data from the other taxa differed from the *A. gambiae* reference. The inferred sequence for each species was aligned with the *A. gambiae* sequences and used for subsequent analyses.

### Genetic differentiation

To estimate levels of genetic differentiation between the strata at the immune genes studied here, we calculated F_ST_ by using Weir and Cockerham’s unbiased estimator (Weir and Cockerham 1984) as implemented in an R script written by Eva Chan (www.evachan.org). F_ST_ was calculated for each gene separately between all pairs of population strata. To determine whether each estimate was significantly greater than zero, we randomly permuted population assignments 10^5^ times, recalculated F_ST_, and asked how many of the randomly permuted data sets exhibited F_ST_ greater than the observed value. Values were considered significantly greater than zero if fewer than 5% of simulations resulted in F_ST_ greater than the observed value.

### Neutrality tests and population genetic statistics

We calculated population genetic statistics for each gene within each population. To identify patterns of genetic variation that are consistent with positive selection, we used a compound statistic called *HEW* (Zeng *et al.* 2007b). This approach combines Fay and Wu’s *H* statistic (Fay and Wu 2000), which is based on site-frequency estimators of the population parameter 4N_e_*μ* while giving extra weight to high frequency derived alleles, with the Ewens-Watterson (*EW*) haplotype based statistic (Watterson 1978) that measures haplotype homozygosity. *HEW* is implemented by calculating *H* and *EW* separately, comparing each test statistic to a null distribution to obtain *p*-values, combining the *p*-values into a vector, and then comparing this vector against empirically determined thresholds to determine whether the vector is consistent with neutrality (Zeng *et al.* 2007b). Empirical thresholds for statistical significance for *HEW* were established by comparing the distribution of *p*-values for the component statistics and finding the threshold that provided the desired statistical cutoff (0.05) for the vector combining the two component *p-*values (Zeng *et al.* 2007b). Null distributions of all test statistics were generated using coalescent simulations conditioned on the number of haplotypes in the sample with the mutation rate set to the population parameter θ (4N_e_*μ*) estimated from the data using Watterson’s estimator (Watterson 1975), as described in Zeng *et al.* (2007b). All coalescent simulations were conservatively conducted with no recombination. The assumption of no recombination is justified because the gene fragments sequenced in the present study are short enough that recombination is not likely to be pervasive in the samples. Accurate estimates of recombination are not yet available in *A. gambiae*, particularly in and around the 2La inversion, and estimating recombination rates from population sequence data can produce inaccurate results (Wall 2000). It should be noted that we used a normalized version of the *H* statistic that was derived by Zeng *et al.* (2006) and was shown to consistently have slightly more power than the original un-normalized version. Because *H* requires an outgroup and *EW* is based on haplotype diversity, the *A. merus* outgroup sequence and *A. gambiae* haplotypes consisting only of silent (synonymous and non-coding) sites were used to calculate the component statistics of *HEW* for each gene and for each population. Calculations of the component statistics of *HEW* and all coalescent simulations were carried out using a program kindly provided by K. Zeng. The resulting *p-*values were corrected for multiple testing using the Benjamini and Hochberg (1995) correction as implemented in the p.adjust function in R (R Development Core Team 2011).

Additional population statistics, including nucleotide diversity, the number of haplotypes, and Tajima’s *D* (Tajima 1989), were calculated. Nucleotide diversity (π) was measured as the average number of nucleotide differences per site using DnaSP v.5.10 (Librado and Rozas 2009). The number of haplotypes, *h*, also was calculated using DnaSP. To test the significance of haplotype and nucleotide diversity for genes with structured genealogies, we simulated neutral genealogies using *ms* (Hudson 2002) conditioned on the empirical clade structure and number of segregating sites in the empirical sample. For each simulated genealogy that satisfied these criteria, we calculated nucleotide and haplotype diversity for each subclade and counted how many simulated genealogies showed values equal to or more extreme than the empirically observed values. Tajima’s *D* was calculated using the software package provided by K. Zeng mentioned above and evaluated for statistical significance in the same simulation framework as for *H* and *EW*.

We also used a multilocus version of the Hudson-Kreitman-Aguade (HKA) test (Hudson *et al.* 1987) to test for deviations from neutral expectations. We used a multilocus version of the HKA test implemented in the program *hka* written by J. Hey (http://genfaculty.rutgers.edu/hey/software#HKA). For this test, we used only variation at synonymous or noncoding sites and *A. merus* as the outgroup (species 2). To determine significance of the observed values and sum of deviations, 10^5^ neutral coalescent simulations were conducted modeled on parameters inferred from the data within the program to establish an empirical distribution of the χ^2^ distribution. Because the comparison is designed for a locus of interest and a “neutral” locus, and we did not sequence any functionally random control loci, we instead compared the focal locus to the nearest upstream and downstream neighbors that did not show a significant *HEW* test statistic.

### Linkage disequilibrium (LD) and haplogroup analysis

We estimated genetic correlation, *r^2^*, between all variant sites within each gene for each population and used this statistic to identify blocks of high LD in genes that rejected neutrality based on the aforementioned *HEW* statistic. *r^2^* was calculated using an R script written by Eva Chan (www.evachan.org) and plotted using the R package LDheatmap (Shin *et al.* 2006). LD plots were visually inspected for all loci with a significant *HEW* result in the homokaryotype groups of GOUNDRY (2La^a^/2La^a^, 2La^+^/2La^+^), and the gene with the most striking LD block was chosen for further analysis. Where we hypothesized that incomplete sweeps or sweeps from standing variation were plausible models in these data, MEGA5 (Tamura *et al.* 2011) was used to calculate and draw neighbor-joining gene trees using the maximum composite likelihood method with uniform substitution rates among sites, and trees were inspected for evidence of distinct clades of genetically similar haplotypes. Alignments were inspected for distinct haplotypes using CodonCode (v3.7), and evidence for increased linkage within and differentiation between groups of haplotypes was used to delineate distinct haplotype groups within the sample. These haplogroups were then designated A and B, and each haplogroup was further analyzed for evidence of positive selection based on the same summary statistics as above.

To test whether the distribution of segregating sites and patterns of nucleotide diversity across the two haplogroups were consistent with the neutral equilibrium model, we conducted an additional 10^5^ coalescent simulations for each gene conditioned on the observed clade structure and the observed number of segregating sites. Specifically, we conducted coalescent simulations under a rejection framework where we retained only simulated genealogies with clade structures similar to the observed genealogy. We then asked how often the simulated data showed values equal or more extreme than those observed in our empirical datasets.

To identify specific regions of high divergence, we calculated Jukes-Cantor corrected divergence (*K*_JC_) using a sliding window analysis in DnaSP with a physical window size of 50 bp and a shift size of 10 bp. After identifying a region of high divergence, we extracted the sequence from both the low and high divergence clades and searched for transcription factor binding sites by comparing the sequences to insect matrices within the TRANSFAC database using the Match 1.0 webserver (BioBase). We set the selection cutoff to minimize false positives and only searched high quality matrices within the insect group.

## Results

### Population differentiation and genetic variation

**Figure 1:**
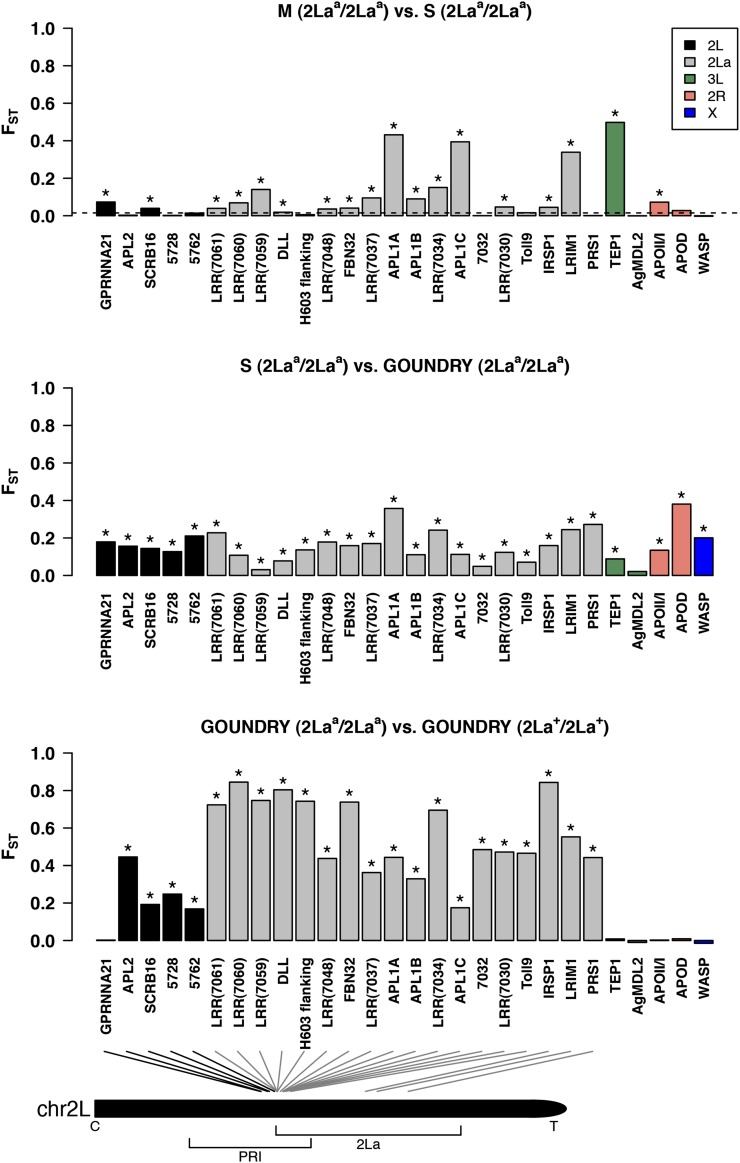
Barplot distributions of genetic differentiation among groups of *A. gambiae* at immune genes. Genetic differentiation was estimated using Weir and Cockerham’s unbiased estimator of F_ST_ between groups of *A. gambiae* at each gene separately. Loci are arranged according to their genomic coordinates with the 2La inversion presented in the inverted arrangement. The vertical bar colors specify genomic region according to the legend. The schematic indicates the physical distribution of the genes on 2L, with C and T representing the centromere and telomere, respectively. The PRI region and 2La are also indicated on the chromosome schematic. Asterisks indicate F_ST_ values significantly greater than zero as determined by permutation tests (see Methods). Both the M and S molecular forms were compared to GOUNDRY separately and exhibited qualitatively similar levels and patterns of differentiation, so only the S-form comparison is presented here.

We examined genetic variation at 28 loci, comprising mostly genes selected by a filtering process designed to enrich for immune-related genes inside the Plasmodium resistance island (PRI) on the *A. gambiae* second chromosome near the proximal boundary of the 2La inversion (Niaré *et al.* 2002; Riehle *et al.* 2006, 2007). Population samples were drawn from three subgroups of *A. gambiae s.s*.: ENDO M and ENDO S forms and the recently discovered cryptic subgroup GOUNDRY (Riehle *et al.* 2011). The genes analyzed here can be grouped by genomic context because many of the genes (18/28) are located inside the large 2La inversion on the left arm of the second chromosome, with many of the genes clustered near the proximal breakpoint of the inversion (Figure 1). Half of the remainder of the genes lie on 2L outside the inversion and the rest are on other chromosomal arms. Twenty of the 28 sequenced loci fell within the PRI (Figure 1). The 2La inversion is nearly fixed for the inverted form in the molecular forms in Burkina Faso, but is segregating at Hardy-Weinberg equilibrium frequencies in GOUNDRY (Riehle *et al.* 2011). When GOUNDRY 2La^+^/2La^+^ and 2La^a^/2La^a^ individuals are contrasted for genetic differentiation, genes in collinear regions of the genome that are physically distant from the inversion show no differentiation among homozygous groups (mean F_ST_ = −0.0005), whereas those inside or near (<2.2 MB) the inversion show extremely high levels of differentiation (mean F_ST_ = 0.52), indicating strong reductions of recombination and independent evolutionary trajectories within and surrounding the 2La inversion (Figure 1). On the other hand, comparisons among the M and S molecular forms and GOUNDRY individuals homozygous for the inverted form revealed relatively constant genetic differentiation across all loci, irrespective of distance from the inversion (Figure 1). M and S molecular forms were compared to GOUNDRY separately and exhibited qualitatively similar levels and patterns of differentiation, so only the S form comparison is presented in Figure 1. These results highlight reductions of interbreeding between the M and S molecular forms and GOUNDRY as well as reductions in recombination between the two forms of the 2La inversion, especially at the breakpoints, and lead us to delineate four groups for subsequent analysis (M, S, GOUNDRY 2La^+^/2La^+^, and GOUNDRY 2La^a^/2La^a^). The contrast between the molecular forms and the GOUNDRY 2La^a^/2La^a^ group also indicates that the origin of the 2La inversion predates the split between all of these groups because differentiation is similar between loci inside and outside the inversion. This pattern is contrary to that which would be expected if the inversion had been introgressed after the subgroups diverged, in which case the inversion might show less differentiation than loci outside the 2La region.

**Figure 2:**
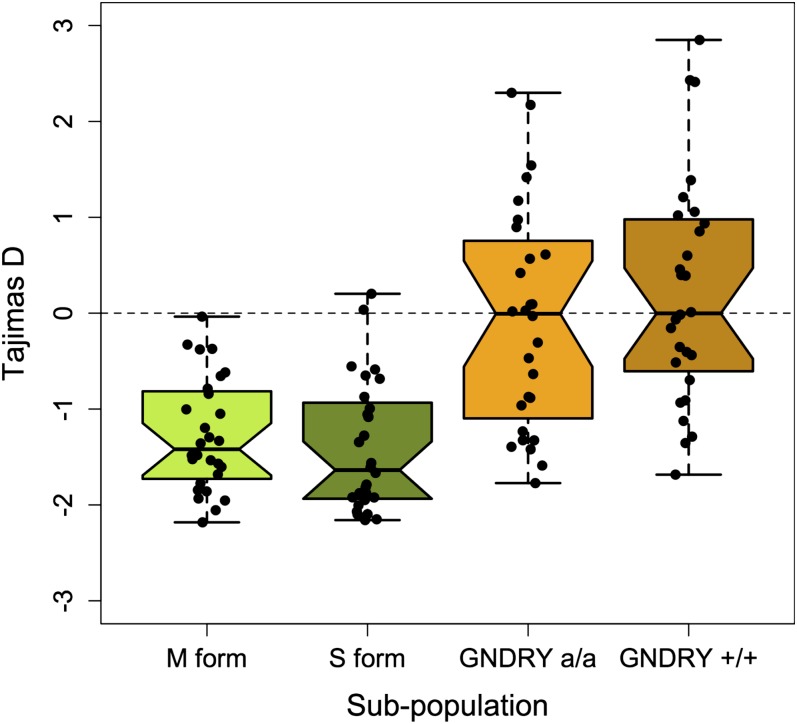
Distribution of Tajima’s *D*. Tajima’s *D* was calculated for all genes using synonymous sites and both boxplots and data points are presented. The dotted line indicates the expected value of *D* under neutral equilibrium population models. GNDRY a/a and GNDRY +/+ refer to GOUNDRY 2La^a^/2La^a^ and 2La^+^/2La^+^, respectively.

Patterns of genetic diversity also differentiate these population strata (Table 1). Levels of synonymous coding variation are 35% lower in GOUNDRY 2La^a^ homokaryotypes (average θ_W_ = 1.64%) compared with M form (θ_W_ = 2.53%) and S form (θ_W_ = 3.66%), indicating that the effective population size of GOUNDRY is substantially smaller than that of the M- and S-form populations, which are distributed across most of West Africa and sub-Saharan Africa, respectively (Lehmann and Diabate 2008). Furthermore, the distributions of allele frequencies differ between GOUNDRY and the M and S forms, possibly indicating distinct demographic histories. Although genes of M and S molecular-form mosquitoes generally have negative values of Tajima’s *D* consistent with recent population growth previously inferred for these populations (Crawford and Lazzaro 2010), GOUNDRY exhibits a distribution of *D* approximately centered on *D* = 0 with a substantially increased variance (Figure 2). The reduced nucleotide diversity and non-negative values of *D* may indicate either a recent bottleneck of at least moderate size and duration in GOUNDRY or that this population has maintained a relatively small and consistent effective population size in the recent past. We also compared levels of variation and distributions of allele frequencies between genes inside and outside of the 2La inversion and found that, while loci associated with the inversion may be more differentiated among inversion forms, levels of diversity and *D* do not differ among genes inside and out of the inversion (Table 1).

**Table 1:** Summary of nucleotide diversity and the site-frequency spectrum among populations of *A. gambiae*

		θ*^b^*		Tajima’s *D**^c^*	
Population	n*^a^*	All	2La*^d^*	All	2La*^d^*
M form	94	0.0253	0.0278	−1.28	−1.24
S Form	136	0.0366	0.0412	−1.41	−1.39
GOUNDRY 2La*^a^*/2La*^a^*	56	0.0164	0.0186	−0.07	−0.13
GOUNDRY 2La^+^/2La^+^	170	0.0114	0.0125	0.22	0.17

a Average number of chromosomes sequenced per gene fragment.

b Average θ calculated for each gene fragment using only synonymous sites.

c Average *D* calculated for each gene fragment using only synonymous sites.

d Statistic calculated using only genes inside 2La inversion.

### Evidence for positive selection

**Figure 3:**
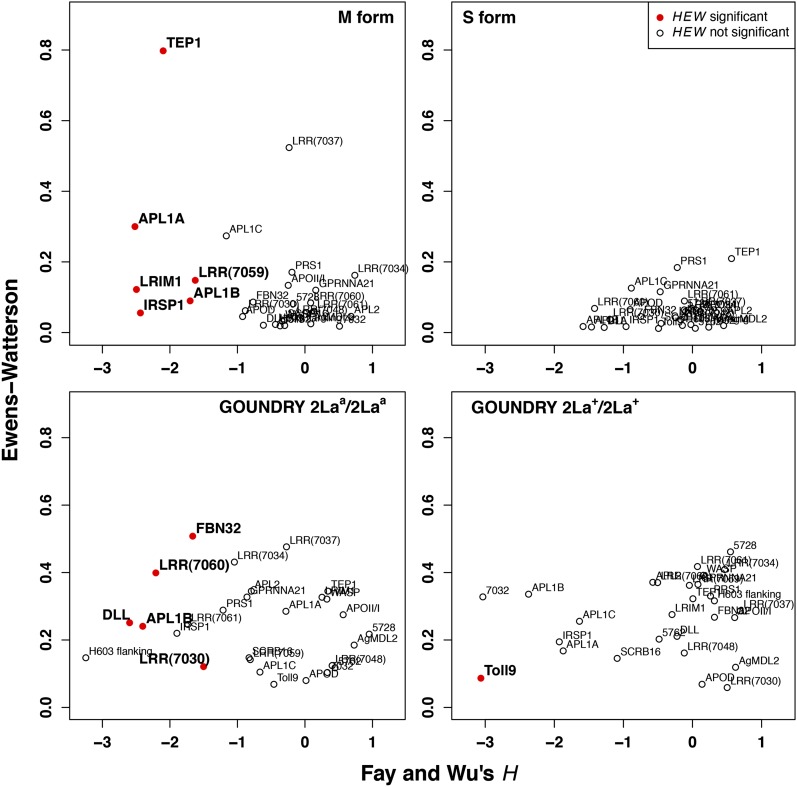
The distribution of Fay and Wu’s *H* and the Ewens-Watterson statistic for all loci in *A. gambiae* populations. Normalized Fay and Wu’s *H* and Ewens-Watterson statistics were calculated on synonymous variation at each gene and evaluated by the use of coalescent simulations (see *Materials and Methods*). Red dots indicate that the *HEW* statistic is significant at a 5% threshold level after correcting for multiple tests. Each panel presents the results for a different group and the gene names are presented next to the corresponding data point.

To determine whether genetic variation at the immune-related genes under study exhibit patterns that are consistent with positive selection, we used summary statistics that measure enrichment of high frequency derived alleles (Fay and Wu 2000) and levels of haplotype homozygosity (Watterson 1978) to ask whether the observed patterns are consistent with neutral evolution. A recent series of papers introduced the *HEW* test that combines Fay and Wu’s site-frequency spectrum based *H* statistic (Fay and Wu 2000) with Ewens-Watterson test for haplotype homozygosity (Watterson 1978) into a compound statistic. The compound statistic *HEW* provides a sensitive and specific approach for distinguishing the footprint of positive selection from both stochastic neutral evolution as well as demographic effects, particularly for short sequence fragments as analyzed here (Zeng *et al.* 2006, 2007a,b). We tested all genes in each of the four groups using *HEW* and, despite the relatively conservative nature of the compound *HEW* statistic, we found putative evidence for positive selection at 11 immune-related genes after correcting for multiple testing (Table 2). Interestingly, of the 11 genes that exhibit evidence for positive selection, only one (*APL1B*) is common to two population strata (Figure 3), implying that the strata reflect ecologically distinct subpopulations whose immune genes are under substantially different selection regimes. The contrast between the molecular forms is remarkable in that evidence for selection was identified at six loci in the M form, but not one gene showed significant departure from neutrality in the S form. Furthermore, there is no overlap among adaptive signals between the two GOUNDRY classes of 2La homokaryotypes (Figure 3), suggesting that the two inversion states experience distinct selective pressure.

**Table 2:** Population genetic summary statistics and test results for loci with a significant *HEW* test corrected *p-*value (statistics for all loci are presented in Table S4)

Locus	n*^a^*	S_syn_*^b^*	*D**^c^*	*H**^d^*	*EW**^e^*	*HEW* corrected *p*-value*^f^*
M form						
* LRIM1*	64	25	−0.1096	−2.4951	0.1221	0.0336
* TEP1*	64	10	−2.1817	−2.0996	0.7979	0.0233
* APL1A*	62	110	−1.1500	−2.5176	0.3002	0.0140
* APL1B*	100	88	−0.5362	−1.7003	0.0898	0.0294
* LRR (7059)*	100	50	−1.8131	−1.6246	0.1480	0.0302
* IRSP1*	64	55	−1.4194	−2.4365	0.0557	0.0140
GOUNDRY 2La*^a^*/2La*^a^*						
* APL1B*	100	70	0.2394	−2.4022	0.2408	0.0252
* LRR (7030)**^g^*	34	22	−1.3252	−1.5011	0.1211	0.0482
* LRR (7060)*	100	38	−2.0531	−2.2083	0.3992	0.0280
* FBN32*	100	15	−1.2392	−1.6624	0.5080	0.0482
* DLL*	100	72	−2.3283	−2.5965	0.2512	0.0252
GOUNDRY 2La^+^/2La^+^						
* Toll9*	100	50	−0.1716	−3.0625	0.0868	0.0224

a Number of chromosomes in the sample. Loci with n = 100 had more than 100 in the original sample, but were down-sampled to 100 for this analysis.

b Number of synonymous segregating sites.

c Tajima’s *D* calculated using only synonymous sites.

d Normalized Fay and Wu’s *H* calculated using only synonymous sites.

e Ewens-Watterson’s haplotype homozygosity statistic calculated using only synonymous sites.

f *HEW p*-value with Benjamini and Hochberg correction for multiple tests. Statistical significance of *HEW* was evaluated by comparison to 10^5^ neutral coalescent simulations of each sample (see Materials and Methods).

g For simplicity of presentation, the unnamed LRR genes are labeled according to a shortened form of their AGAP identifier.

The *HEW* statistic is largely unaffected by population growth and has limited sensitivity to population bottlenecks, particularly as compared to Tajima’s *D* or Fay and Wu’s *H* (Zeng *et al.* 2007b). Despite these advantages, false positive results can occur if haplotype reconstruction is incorrect (Zeng *et al.* 2007a). To rule out this potential source of false positive test results, we evaluated the phase inference results based on several criteria to determine whether genes with significant *HEW* statistics also showed relatively low confidence phase reconstruction. When the statistical software PHASE assigns heterozygous sites to haplotypes, confidence probabilities are calculated for each site that reflect the degree of statistical confidence in the assignment, where a probability of one reflects no ambiguity and 0.5 indicates complete ambiguity (Stephens *et al.* 2001). In our dataset, more than half of inferred sites were assigned to a haplotype with a confidence probability of one, thanks in part to the power of haplotype inference achieved with large numbers of individuals sampled and small sequence windows with little recombination. There is some uncertainty in haplotype reconstruction based on the remaining sites with probabilities less than one, and we sought to use the information contained in these probabilities to evaluate the possible effects of this uncertainty on our *HEW* results. We evaluated each run of PHASE (see *Materials and Methods*) based on the proportion of sites that were inferred or imputed as well as the distribution of confidence probabilities, reasoning that haplotype reconstruction might be most problematic in genes with relatively more missing and heterozygous sites and, therefore, more low confidence probabilities. To determine whether this was a concern with respect to our inferences of positive selection, we first evaluated genes based on the proportion of the total sequence that was phased or imputed. None of the genes that rejected neutrality based on *HEW* were in the top 5% for proportion of either phased or imputed sites (Table S5). We then ranked the genes by proportion of variant sites at which the confidence probability estimated by PHASE was less than one. One of the genes that rejected neutrality, *LRR(7030)* (for simplicity of presentation, the unnamed LRR genes are labeled according to shortened forms of their AGAP identifiers) in GOUNDRY 2La^a^/2La^a^, was in the 5% tail (Table S5). This finding suggests that the results from this gene should be interpreted with caution. Generally speaking, however, the mean confidence probability among sites with probabilities less than one was 0.78 at this locus. Although it is difficult to fully evaluate the success of the phase inference process in the absence of experimental validation, because the genes that show evidence of positive selection are not among those with the lowest confidence or even those that required the most phasing, we do not believe that phasing errors are likely to be causing false positives in our tests for positive selection.

### *TEP1*, *LRIM1*, and *APL1*

**Figure 4:**
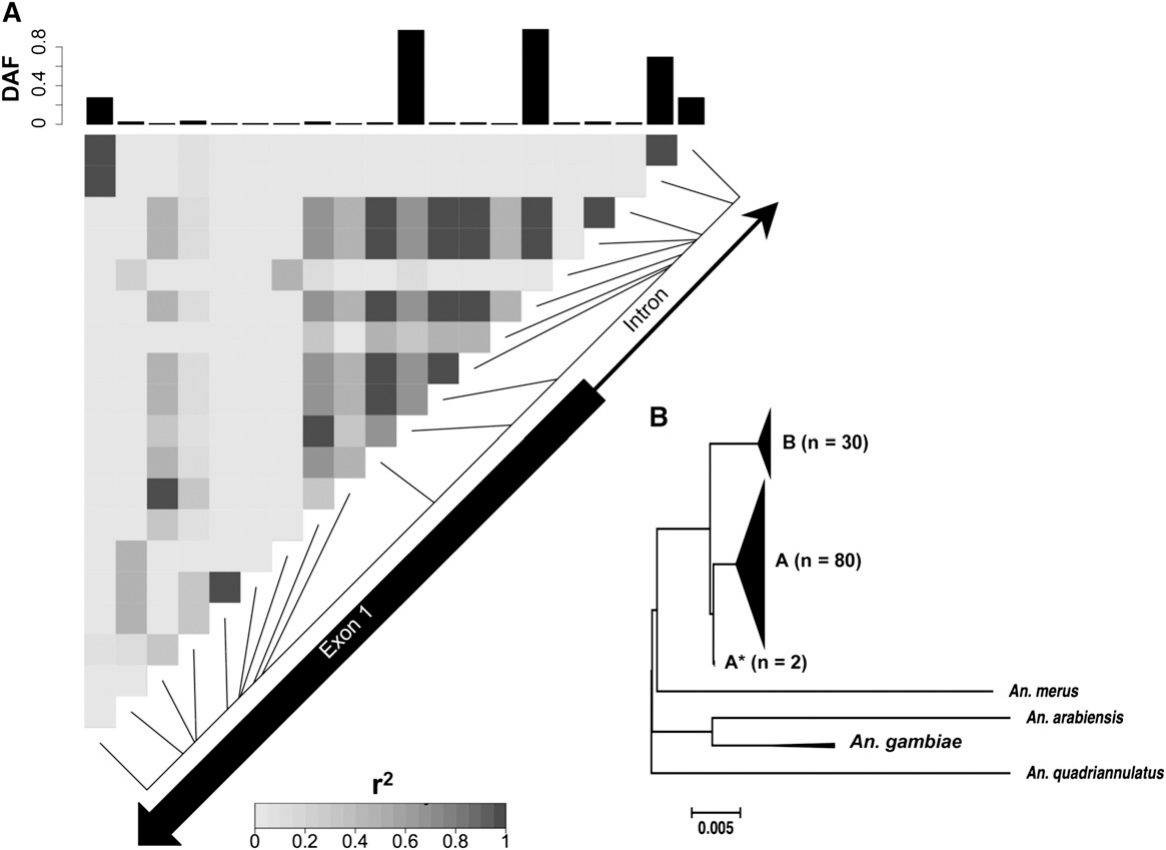
LD plot and neighbor-joining tree of *FBN32* in GOUNDRY 2La^a^/2La^a^. (A) Linkage disequilibrium (r^2^) plotted among variant sites in the sequenced fragment of *FBN32*. Each pixel represents an r^2^ value according to the shade of gray, as indicated in the scale. Greater r^2^ values indicate increased linkage among those sites. The exon structure of *FBN32* is placed on the diagonal of the plot to indicate the physical location of each variant site. The frequency of the derived allele frequency (DAF) relative to *A. merus* is plotted in the barplots above and to the side of the LD plot. (B) Neighbor-joining tree of all *FBN32* sequences from GOUNDRY 2La^a^/2La^a^ as well as three additional taxa: *A. merus*, *A. arabiensis*, and *A. quadriannulatus*. The scale bar indicates genetic distance. Large clades of genetically similar taxa were collapsed for presentation.

An important validation of our analysis was the recovery of signals of positive selection in two genes previously indicated as evolving adaptively through analysis of independent datasets and analytical approaches. Positive selection has been identified at both *TEP1* (Cohuet *et al.* 2008; Obbard *et al.* 2008; White *et al.* 2011) and the *APL1* gene cluster (Rottschaefer *et al.* 2011) in the M-form population, and our new analysis indicated adaptive evolution at both of these loci (Figure 3). In addition, it has been speculated that, since a physical complex is formed between the proteins encoded by *TEP1* and *APL1C* as well as a third protein (LRIM1), the patterns of variation at *TEP1* and the *APL1* locus may reflect coordinate adaptive evolution (Fraiture *et al.* 2009; Povelones *et al.* 2009; Rottschaefer *et al.* 2011). Thus far, no signals of adaptive evolution have been identified at *LRIM1* in *A. gambiae* (Obbard *et al.* 2007; Slotman *et al.* 2007; Cohuet *et al.* 2008), but our new analysis points to an enrichment of high-frequency-derived alleles (*H_norm_* = −2.49; uncorrected *p* = 0.0222) and an increase in haplotype homozygosity (*EW* = 0.12; uncorrected *p* = 0.0513) at *LRIM1* in the M-form population that is inconsistent with neutral evolution (*HEW* corrected *p* = 0.0336). We also find evidence for positive selection at the *TEP1* locus (*HEW* corrected *p* = 0.0233), consistent with coordinate adaptive evolution among the proteins making up the complex. When we analyze the *APL1* paralogs separately, we find that *APL1C*, the only *APL1* paralog involved in the described protein complex, is a clear outlier from the majority of loci in this population (Figure 3), although its *HEW* statistic is marginally nonsignificant after multiple testing (*HEW* corrected *p* = 0.084). If selection is acting on the three members of the complex in a coordinate fashion, *TEP1* is an outlier (Figure 4) suggesting that selection is stronger on this locus than on *LRIM1* and *APL1C*.

### FBN32

Of all genes that showed a significant departure from the neutrality by the *HEW* statistic, *FBN32* (AGAP007041; *HEW* corrected *p* = 0.0482) in the GOUNDRY 2La^a^/2La^a^ subgroup showed the most striking pattern of linkage disequilibrium (Figure 4). Inspection of the sequence data revealed three derived SNPs in perfect linkage disequilibrium at the boundaries of the sequenced fragment. These SNPs mark two distinct major haplotype clades, hereafter referred to as haplogroups A and B, the larger of the two also harboring two possible recombinant haplotypes (clade A*; Figure 4). This genealogical structure is significantly unlikely under a neutral model (*p =* 0.0062), and we hypothesized that the presence of two sharply defined clades could be consistent with either a partial selective sweep (Hudson *et al.* 1997), a sweep from standing genetic variation (Przeworski *et al.* 2005), or a classical selective sweep with at least one recombination event occurring during the sweep.

Several lines of evidence suggest that an incomplete sweep cannot explain the data. First, if we assume that the less-variable B haplotype is the selected haplotype, it would be segregating at a relatively low frequency in the population (27% or 30 of 110 chromosomes) that the *HEW* test statistic has very low power to detect (Zeng *et al.* 2007b), implying that the deviation from neutrality detected by the *HEW* test stems from the entire sample instead of just one clade. Second, under a partial selective sweep model, we would expect the selected clade to lack variation whereas the other clade harbors presweep variation, but this is not what we find. Comparison with simulated neutral genealogies indicates that the low average number of pairwise differences (π_A_ = 0.0021; π_B_ = 0.0002) is significantly unlikely under the neutral model in both clades (clade A *p <* 0.0005; clade B *p <* 0.05; Table 3). Moreover, when applied to the larger (n = 80 chromosomes) and more diverse A clade, neutrality tests (*D*, *H*, *EW*, *HEW*) reject the neutral model (all *p <* 0.05; Table 3).

**Table 3:** Population genetic summary statistics for haplogroups of *FBN32* in GOUNDRY 2La^a^/2La^a^ and *Toll9* in GOUNDRY 2La^+^/2La^+^

Clade	*n**^a^*	*h**^b^**^,^**^c^*	S*^d^**^,^**^c^*	π*^e^**^,^**^c^*	*D**^f^*	*H*^g^*^,^**^h^*	*EW*^i^*^,^**^h^*	*HEW**^j^**^,^**^h^*
*FBN32*								
All	110	10	18	0.0046*	−1.2392	−1.6624	0.508	**
A	80	8 ^NS^	17	0.0021***	−2.16**	−2.60*	0.81**	**
B	30	2 ^NS^	1**	0.0002*	NA	NA	NA	NA
*Toll9*								
All	122	42	42	0.0183^NS^	−0.1716	−3.0625**	0.0868*	***
A	20	3 ^NS^	3**	0.0013*	NA	NA	NA	NA
B	102	39 ^NS^	39	0.0001***	−1.4	−1.79*	0.09**	**

NA, not available.

a Number of chromosomes in each clade.

b Number of haplotypes in each clade.

c For *h*, S, and π, statistical significance was evaluated by comparison to 10^5^ coalescent simulations conditioned on clade structure (see *Materials and Methods*).

d Number of segregating sites in each clade.

e Per site nucleotide diversity calculated on all segregating sites.

f Tajima’s *D* calculated on synonymous sites.

g Normalized Fay and Wu’s *H* calculated on synonymous sites.

h For the neutrality tests, statistical significance was evaluated by comparison to 10^5^ neutral coalescent simulations of each sample sub-set/clade (see *Materials and Methods*). Statistical significance is indicated as * < 0.05, ** < 0.005, and *** < 0.0005.

i Ewens-Watterson’s haplotype homozygosity statistic calculated on synonymous sites.

j *p*-value of the *HEW* test corrected for multiple tests.

Collectively, these results confirm that the A and B clades both harbor patterns of genetic variation that are inconsistent with neutrality; thus, the data are more consistent with a complete sweep with recombination rather than an incomplete sweep. To rule out the possibility that this locus could have an unusually low mutation rate that could be driving these results, we compared patterns at *FBN32* to its nearest neighbors in the dataset using an HKA test (Hudson *et al.* 1987), and found that *FBN32* harbors significantly fewer polymorphisms and is significantly more diverged from *A. merus* than expected under neutral model (uncorrected *p* = 0.0176).

The division of the haplotypes into two large clades could have arisen due to either the presence of the selected site on two chromosomal backgrounds before selection or a recombination event could have occurred during the selective event. It is difficult to distinguish between these two models. Overall, the data are consistent with a model of positive selection at *FBN32* in this population that may have involved a sweep with recombination or selection on standing variation.

### Toll9

**Figure 5:**
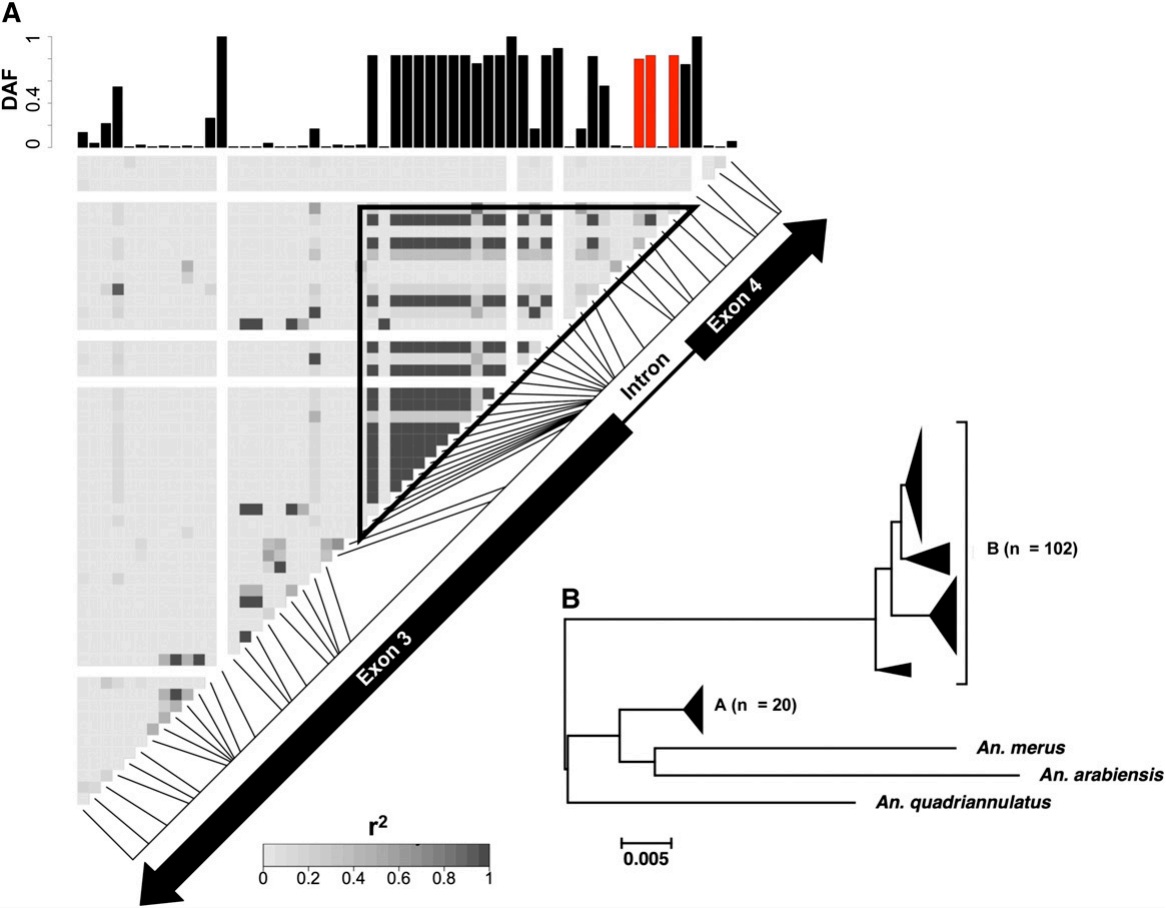
LD plot and neighbor-joining tree of *Toll9* in GOUNDRY 2La^+^/2La^+^. Linkage disequilibrium (*r*^2^) plotted among variant sites in the sequenced fragment of *Toll9*. Each pixel represents an *r*^2^ value according to the shade of gray, as indicated in the scale. Greater *r*^2^ values indicate increased linkage among those sites. The exon structure of *Toll9* is placed above and beside the plot to indicate the structural location of each variant site. The frequency of the derived allele relative to *A. merus* is plotted in the barplots above and to the side of the LD plot. The red bars indicate the three nonsynonymous sites and the triangle delineates the block of linked sites. (B) Neighbor-joining tree of all *Toll9* sequences from GOUNDRY 2La^+^/2La^+^ as well as three additional taxa: *Anopheles merus*, *Anopheles arabiensis*, and *Anopheles quadriannulatus*. The scale bar indicates genetic distance. Large clades of genetically similar taxa were collapsed for presentation.

**Figure 6:**
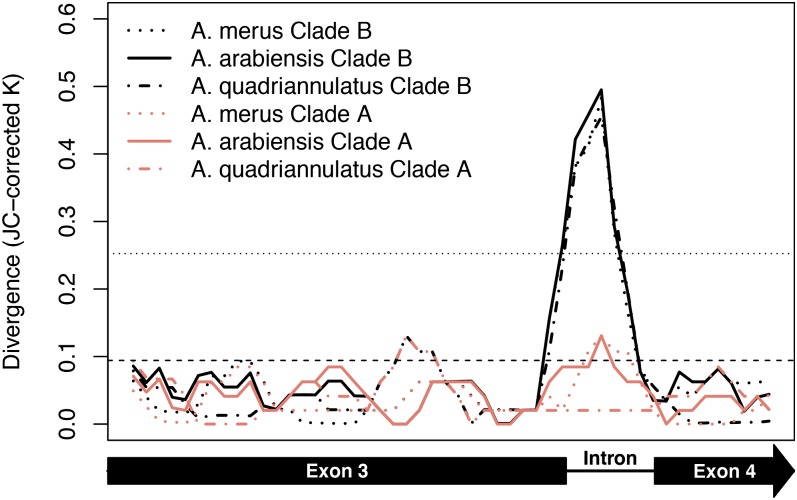
Clade specific patterns of divergence at *Toll9* intron. Sliding window analysis of Jukes-Cantor corrected divergence (*K*_JC_, range 0 to 1) at all sites relating the two haplotype groups (A and B) to three sister species. Divergence was calculated for 50-bp physical windows shifting 10 bp for every consecutive window. The top horizontal dotted line indicates the average maximum divergence per window for *Toll9* in the M and S molecular forms as well as GOUNDRY 2La^a^/2La^a^. The lower horizontal dashed line indicates the average maximum divergence per window for all other sequenced loci that included an intron in the GOUNDRY 2La^+^/2La^+^ population. The legend indicates the color and line style for each clade/sister taxa comparison. The schematic under the plot depicts the exon structure in the sequenced region of *Toll9*.

In the GOUNDRY 2La^+^/2La^+^ group, the only gene to show significant evidence for non-neutral evolution based on the *HEW* statistic was *Toll9* (Figure 3). Despite showing a significant departure from neutrality, this gene harbors substantial variation in this subpopulation (θ_W_ = 0.0204), signaling that these data are not consistent with a recent and strong selective sweep. Similarly to the pattern observed in *FBN32* in GOUNDRY 2La^a^/2La^a^, two distinct clades are present in the *Toll9* data, a genealogical structure that is significantly unlikely under a neutral model (*p* < 10^5^; Figure 5). Analysis of LD in this region reveals an LD block consisting of 18 sites in linkage disequilibrium separating the two clades, three of which are nonsynonymous substitutions in the fourth exon (Figure 5). Of these sites, 17 are fixed between the two clades. A neighbor-joining tree reveals an interesting and unexpected topology where the A clade and the sister taxa are separated from a long branch from the B clade sequences (Figure 5). Plotting divergence across the sequence for each clade separately reveals a large spike in divergence between clade B and the sister taxa (*K*_JC_ = 0.495) restricted to the intronic sequence, suggesting that introgression from these sister taxa cannot explain the spike in divergence (Figure 6). We found that divergence from the sister taxa (*K*_JC_) never exceeded 0.155 in similarly sized sequence windows from other genes in our data set, confirming that the *Toll9* sequence is an outlier. We also examined divergence at *Toll9* in other subpopulations and found similar, albeit smaller spikes in the intronic sequence (maximum 50bp window *K*_JC_ = 0.322 in M-form population, mean across all populations *K*_JC_ = 0.252), suggesting that both haplotype groups existed and were segregating at intermediate frequency before the split of GOUNDRY from the M and S molecular forms. We considered that the unusual B clade could have arisen through a paralogous gene conversion event, for example with another member of the *Toll* family. To test this hypothesis, we used BLAST to search the clade B sequence against the *A. gambiae* genome and the NCBI nr sequence database, but we found no significant matches to any other available sequence other than existing *A. gambiae Toll9* sequences. It is possible that the sequence may have been introgressed from a species not sampled in this study, but we have no data to support or refute that hypothesis.

The divergent sequences defining clades A and B may represent a functional balanced polymorphism. Among other possible functions, introns can harbor transcription-binding sites that affect expression patterns of the surrounding gene, or even a different gene in *trans*. We compared the intronic sequence from both haplotype groups to known insect transcription factor binding site motifs in the TRANSFAC database and found that both haplotypes showed approximately equal matches to three binding motifs (BR-C z1, Hairy, Elf-1). Each haplotype also showed matches to at least one unique motif. The divergent B haplotype showed a match (matrix match = 0.852) to a motif that recruits the NF-kappaB transcription factor Dorsal that has been shown in Drosophila to function both in dorsal-ventral patterning during embryogenesis as well as in activating an immune response as a component of the Toll signaling pathway (Lemaitre *et al.* 1995; Ghosh *et al.* 1998; De Gregorio *et al.* 2002). The homolog of Dorsal in Anopheles, Rel1, has been shown to play a role in driving immune responses against a variety of pathogens, mediated at least in part through the action of the APL1C and TEP1 proteins (Barillas-Mury *et al.* 1996; Frolet *et al.* 2006; Mitri *et al.* 2009), so it is tempting to speculate that this motif may serve a role in immunity, but further experimental analysis is required to determine any functional differences between the divergent haplotypes.

We propose similar population genetic models to explain the *Toll9* and *FBN32* data: a partial selective sweep or a selective sweep with recombination either from standing genetic variation or with subsequent recombination during the sweep. Under the partial selective sweep model, the selected haplogroup would be expected to show a departure from neutrality whereas the alternative haplogroup would show patterns of genetic variation consistent with neutral expectations. To determine whether the *Toll9* data fit these expectations, we analyzed the haplogroups separately for evidence of non-neutral evolution. Population genetic analysis of the B haplogroup indicates a significant departure from neutrality in this clade, reflecting a scooped shape in the site-frequency spectrum enriched in both rare and high frequency derived sites (*H =* −3.0625; *p* < 0.005; Table 2). The A haplogroup, however, also exhibited a significant paucity of genetic variation compared to neutral expectations (*p* < 0.005), suggesting the possibility of recent positive selection acting to remove linked variation in this clade, but the very low level of polymorphism (three segregating sites) precludes further analysis of this clade with neutrality tests (Table 2). The alternative hypothesis of selection on standing variation predicts that the selected allele was segregating at appreciable frequencies in the population on multiple backgrounds at the time of the selective event. Under this model, we would expect to find private fixations in this population as well as chromosomes bearing both genetic backgrounds segregating in other populations. Although the degree of linkage disequilibrium is much lower in other populations, the SNPs that delineate the two clades in GOUNDRY 2La^+^/2La^+^ are segregating at intermediate and even high frequencies in the M and S molecular forms as well as GOUNDRY 2La^a^/2La^a^ (data not shown). Furthermore, comparison with the outgroup species *A. merus* reveals 4 derived fixations that are unique to GOUNDRY 2La^+^/2La^+^, three of which are synonymous substitutions and the fourth of which falls within an intron (Figure 5).

Although these fixations and deficits of diversity could reflect sites linked to an adaptive fixation as expected under a sweep model, they could also be a feature of *Toll9* residing inside the polymorphic and highly diverged 2La inversion. We tested this hypothesis by comparing *Toll9* to its neighbors (*LRR(7030)* and *IRSP1*) using a multilocus HKA test (Hudson *et al.* 1987) and found that, although the data were significantly inconsistent with equivalent evolutionary rates across the genes (*p* < 0.0125), the deviation is largely driven by excess divergence and lower than expected polymorphism at *IRSP1*, whereas the *Toll9* data more closely fit expected levels of divergence and polymorphism. The results of this test support the neutral model for *Toll9*, contradicting the *HEW* test result. However, this test may be inappropriate for these data because the diverged intronic sequence may have been a single mutational event and reflect a balanced polymorphism that could reduce the rate of fixation at this locus, in turn downward biasing the estimate of the mutation parameter 4N_e_μ in the HKA model reducing the power to detect reductions in polymorphism. In both intraspecific and interspecific comparisons, the presence of the highly diverged intronic haplotypes makes these data somewhat difficult to interpret. Nonetheless, both global and clade-specific summary statistics point to positive selection at this locus (Table 3), and functional studies of *Toll9* are needed to identify both the selective agent as well as the functional role, if any, of the diverged intronic haplotypes.

## Discussion

We sequenced alleles from 28 immune-related loci in wild samples of multiple genetic subpopulations of *A. gambiae*, obtaining unprecedented sample sizes and providing the first opportunity to contrast patterns of molecular evolution at immune-related loci in the recently discovered GOUNDRY subgroup (Riehle *et al.* 2011) with those in the indoor-resting M and S molecular forms. Analyses of haplotypic and genetic diversity revealed sharp differences among these strata in levels of genetic diversity and allele frequencies in coding sequence, as well as evidence for significant deviations from neutrality at 11 loci among these populations. Further experimentation will be necessary to determine the nature of the selective pressures behind the signals observed here. Our results do, however, allow some speculation on the distribution and nature of the selective events affecting these loci.

### Selection across functional classes

Our results reveal possible evidence for positive selection at genes coding for proteins with a broad range of functions. In one case, we found evidence for positive selection at the developmental morphogen *Distal-less (DLL)*. Our previous studies showed a highly significant association between microsatellite (H603) alleles inside an intron of *DLL* and infection by *P. falciparum* (Niaré *et al.* 2002; Riehle *et al.* 2006), and the signal identified here may reflect linked selection on the functional site driving these signals, although the genetic mapping was conducted in the M and S molecular forms while the signal detected here came from GOUNDRY, where such mapping has not yet been done. However, we also sequenced an intronic fragment flanking H603 and did not find evidence for selection, highlighting the need for further analysis of this genomic region to identify the linked functional site(s) identified in the previous genetic mapping studies. In another case, the gene encoding FBN32 (also known as FREP39), a member of a pathogen recognition receptor family in invertebrates (Gokudan *et al.* 1999; Dong and Dimopoulos 2009), also showed possible evidence for selection. Expression analyses of this gene revealed expression patterns restricted to the abdomen and midgut, the larval salivary gland, and male accessory glands (Dong and Dimopoulos 2009; Neira Oviedo *et al.* 2009; Baker *et al.* 2011). With respect to immune function, *FBN32* was up-regulated in response to immune challenge with a gram-negative bacteria, a fungal pathogen, and *P. falciparum* but not the rodent malaria parasite *Plasmodium berghei* (Dong and Dimopoulos 2009), indicating some degree of generality in its immune function.

Leucine-rich repeat (LRR)-containing proteins, a superfamily composed of 180 proteins in *A. gambiae* (Waterhouse *et al.* 2010), featured prominently among the original filtered gene set within the PRI and also among the genes that showed possible evidence for selection. In fact, the PRI locus as a window contains the largest number of LRR genes in the *A. gambiae* genome (Riehle *et al.* 2006). One class of LRRs, Toll-like receptors (TLRs), are *trans*-membrane proteins known to act as pathogen recognition molecules in mammals (Means *et al.* 2000). Strong functional evidence is available for only a small number of TLRs in insects, and these TLRs have roles in development and immune-related signal transduction (Imler and Hoffmann 2001; Imler and Zheng 2004). We found evidence for positive selection acting on the TLR *Toll9* in the GOUNDRY subpopulation. The exact functional role of *Toll9* is unknown, but in several studies other authors have shown that *Toll9* is slightly up-regulated after a bacterial immune challenge in larvae and expression is concentrated in the midgut of adults, particularly after a blood-meal (Luna *et al.* 2002; Marinotti *et al.* 2005; Baker *et al.* 2011). Phylogenetic comparisons of the *Toll* genes may provide insight into protein function in that *A. gambiae Toll9* clusters with mammalian TLRs based on its ectodomain structure and sequence similarity of the intracytoplasmic domain, suggesting that it may be an ancestral TLR in insects (Du *et al.* 2000; Imler and Zheng 2004; Waterhouse *et al.* 2007).

The other LRRs that showed evidence for positive selection included several more characterized LRRs (*APL1B*, *APL1A*, and *LRIM1*) as well as three uncharacterized LRRs (*LRR(7030)*, *LRR(7059)*, *LRR(7060)*). Interestingly, structural similarities between APL1 and LRIM1 proteins prompted a bioinformatic search through the *A. gambiae* genome for genes that code for other LRIM-like proteins that turned up 24 candidates, but the three uncharacterized LRRs studied here were not among them (Waterhouse *et al.* 2010), implying yet another subclass of LRRs in mosquitoes. Collectively, our results point to selection acting on proteins with a broad range of putative immune function, most of which show little specificity with respect to the pathogen classes to which they respond. This observation is consistent with gathering evidence supporting a model wherein selection pressures derive from a diverse suite of pathogens, including those that infect larvae, driving the evolution of a generalized immune response (Rottschaefer *et al.* 2011; Mitri and Vernick 2012).

### No evidence for Plasmodium-driven selection

Our focus on candidate genes within the PRI provides the potential opportunity to identify Plasmodium-driven selection pressure on Anopheles immune genes because our candidate ascertainment process intentionally enriched for immune-related genes that may play a role in resisting Plasmodium infection on the basis of experimental and bioinformatic evidence (Riehle *et al.* 2006). The epidemiological importance of GOUNDRY is not currently known, although in experimental infections, this population is physiologically more permissive than M or S form *A. gambiae* to infection with *P. falciparum* (Riehle *et al.* 2011). Because the M and S molecular forms are both primary malaria vectors in sub-Saharan Africa, and rates of natural *P. falciparum* infection in wild M and S mosquitoes are equivalent (Wondji *et al.* 2005; Ndiath *et al.* 2008; Trout Fryxell *et al.* 2012), Plasmodium-driven selective pressure, if it exists, should be shared among these subgroups. However, in our data, only 1 of the 11 signals of positive selection (that at *APL1B*) was shared by two subpopulations (Figure 3), and we found no evidence of non-neutral evolution in any genes in the S-form population. Thus, the absence of shared selection signals among population subgroups that are epidemiologically equivalent for *P. falciparum* infection suggests that Plasmodium itself is not the main agent driving the evolution of these genes. Differences in sample sizes or the number of variant sites among populations could lead to reductions in statistical power that could generate false-negative results, but this is not likely to explain the observation because the populations with the fewest signals of possible selection (S form and GOUNDRY 2La^+^/2La^+^) had larger sample sizes in most cases than both of the populations that did show signals of putative natural selection (Table S4).

Alternatively, the selection pressures driving non-neutral evolution at these loci could be related to differential pathogen exposure associated with ecological differences among these populations. Although some of the immune genes studied affect susceptibility to infection by *P. falciparum* in laboratory gene-silencing experiments, some of these loci appear to also play a role in resistance to bacteria and non-human malaria parasites. On this model, selective pressure driven by other environmental pathogens, acting on immunity related genes, shapes mosquito immune mechanisms that can also be addressed against *P. falciparum*, in turn enhancing or decreasing malaria transmission. In fact, the increased susceptibility of GOUNDRY to malaria parasite infection (Riehle *et al.* 2011) could be explained by such a mechanism, under the hypothesis that a change of ecological niche in this population has shaped a new pattern of selection in the immune genes.

### Niche specialization

The striking division of selective pressures among populations points to a model of recent ecological niche specialization. Specifically, our results are consistent with a model in which the M form and GOUNDRY are moving into novel ecological niches, exposing genetic variation to novel selection pressures in the new environments. Under this model, the genetic variation segregating in GOUNDRY and the M form would be expected to be largely a subset of S-form variation. As expected under this model, the majority of segregating sites in both the M form and GOUNDRY 2La^a^/2La^a^ are shared with the S form (mean proportion of M and GOUNDRY 2La^a^/2La^a^ sites shared with S form = 0.5892 and 0.6309, respectively, after correction for sample size), but the opposite is not the case (proportion of S-form sites shared with M form = 0.4729 and with GOUNDRY 2La^a^/2La^a^ = 0.3564, after correction for sample size). Indeed, this model has been proposed previously to explain the relationship between the M and S forms based on the ecological observation that the M form exploits human-derived marginal habitats (della Torre *et al.* 2002; Costantini *et al.* 2009; Simard *et al.* 2009), and to explain molecular data indicating lower levels of genetic diversity (Cohuet *et al.* 2008) as well as more recent population growth in the M form relative to the S form (Crawford and Lazzaro 2010).

Although adaptive divergence at immune genes is not likely to drive the speciation and niche specialization process, exploitation of novel environments is likely to be accompanied by novel pathogen pressures, potentially leading to adaptation of immune factors as observed here (Lee 2002). For example, the more permanent, disturbed, human-derived larval habitats preferred by the M-form population harbor more abundant and complex insect communities (Diabaté *et al.* 2008; Gimonneau *et al.* 2010, 2012), and could also include a greater diversity of pathogens. The lower genetic diversity of GOUNDRY relative to the M and S molecular forms, and the fact that genetic variation in GOUNDRY, like M, is largely a subset of that in the S molecular form, suggests that GOUNDRY is also a derived population that could be moving into novel environments.

As in any molecular population genetic analysis of natural selection, it is impossible to state conclusively the selective agent responsible for the observed patterns. However, it seems reasonable in this case to exclude the human malaria parasite *P. falciparum* as the driving force behind the signals detected in our data, considering the striking division of selective signals among population strata. A compelling alternative explanation is that pathogens in the larval habitats may be driving evolution of these immune genes, and this may have implications for malaria transmission. If broad-spectrum immune factors involved in responding to multiple pathogen classes, one of which including *P. falciparum* (Meister *et al.* 2005, 2009; Dong *et al.* 2006, 2009), are evolving in response to non-Plasmodium pathogens, susceptibility to the malaria parasite could be affected. Many of the immune proteins studied here (*e.g.*, FBN32, LRR(7060)) have not been thoroughly tested functionally for anti-Plasmodium activity. But almost all of the loci that show putative signals of selection are within the PRI region that showed significant association to Plasmodium resistance phenotypes, and these proteins warrant further genetic and functional analysis.

In 1949, Haldane hypothesized, with little knowledge of the underlying molecular mechanisms, that host−pathogen interactions must be an important factor in shaping the ecological patterns observed in nature (Haldane 1949). In this study, we tested candidate immune-related genes for evidence of this evolutionary conflict and found a striking pattern that provides grounds to make the reverse inference from molecular evolution to ecology. Specifically, the distribution of putative pathogen-related signals of selection among populations of *A. gambiae* implies that these populations occupy distinct ecological niches and correspondingly experience disparate host-pathogen interactions.

## Supplementary Material

Supporting Information
