# Supplementary material for: Evidence for Population-Specific Positive Selection on Immune Genes of Anopheles gambiae
Source: G3 (Bethesda). 2012 Dec 1;2(12):1505–19. doi: 10.1534/g3.112.004473 (PMC3516473; doi:10.1534/g3.112.004473)
Supplement: Supporting Information [file supp_2_12_1505__index.html]

Supporting Information 

# Evidence for Population-Specific Positive Selection on Immune Genes of *Anopheles gambiae*

## Supporting Information for Crawford *et al.*, 2012

**Files in this Data Supplement:**

- Supporting Information - Tables S1-S5 (PDF, 162 KB)
- Table S2 - Fragment and PCR Oligo information (PDF, 81 KB)
- Table S3 - Sequenced fragment physical genomic locations (PDF, 76 KB)
- Table S4 - Population genetic summary statistics for all genes in each population (PDF, 128 KB)
- Table S5 - Results from *post-hoc* evaluation of haploype reconstruction for genes with significant *HEW* result (PDF, 85 KB)
- Table S1 - Mosquito population assignment, molecular form, and 2La karyotype (.xlsx, 26 KB)
