# Supplementary material for: Evidence for Population-Specific Positive Selection on Immune Genes of Anopheles gambiae
Source: G3 (Bethesda). 2012 Dec 1;2(12):1505–19. doi: 10.1534/g3.112.004473 (PMC3516473; doi:10.1534/g3.112.004473)
Supplement: Supporting Information [file supp_2.12.1505_004473SI.pdf]

Table S1 Mosquito population assignment, molecular form, and 2La karyotype.

Table S1 is available for download as a Microsoft Excel spreadsheet at

http://www.g3journal.org/lookup/suppl/doi:10.1534/g3.112.004473/-/DC1.

Each row in the spreadsheet corresponds to an individual mosquito. Columns indicate mosquito identifier, the malaria transmission season in which the mosquito was collected, the molecular form (see Methods), and karyotype of the 2La chromosomal inversion (see Methods).

Table S2 Fragment and PCR Oligo information.

Identifier	Gene	PCR fragment	Forward oligo	Reverse oligo	Fragment Length	Year Sampled
AGAP005681	GPRNNA21	AmS010b	5'-GCATCATCATCGGTCACCG-3'	5'-CTGAGTCACCTGCAAACCG-3'	528	2007
AGAP005693	APL2	AmS011b	5'-CTATCCACCGTCCAGTTTG-3'	5'-GGTTCGGTGGAATTCTAACC-3'	561	2007
AGAP005716	SCRB16	AmS013a	5'-TGCCGAAGATGAAACGTACG-3'	5'-CGTGCTAAAGATTGTCATCCG-3'	534	2007
AGAP005728	5728	AmS048b	5'-GAATTGCGCAAACAGTCCAG-3'	5'-CACGTTCGATATCTCGCTGA-3'	650	2007
AGAP005762	5762	AmS049b	5'-ATCGATGTCCTCGGCACTAC-3'	5'-GATGGTCAAAGCCAACGAAC-3'	525	2007
AGAP006102	PRS1	AmS052b	5'-CGAAAGTGATTCCGGACAAG-3'	5'-TTATCGCTCGCACAGCAC-3'	304	2007
AGAP006348	LRIM1	AmS059e	5'-CCTCGTACCGCTTGACGAT-3'	5'-GTGACCTGGATCAGTCTGC-3'	576	2007
AGAP006421	IRSP1	AmS053a	5'-ATGGCCATCTGGATAGCTTG-3'	5'-GATATTCGCTCCACCAGCTC-3'	563	2007
AGAP006974	TOLL9	AmS001b	5'-GCATCTCGAACTGACACCAG-3'	5'-TTCGGATATTCCGGAGGAG-3'	536	2007
AGAP007030	LRR(7030)	AmS002a	5'-AGAAACAACAGTCGCAAGCTC-3'	5'-ACATGCTGTGCACCATAAAGAA-3'	501	2007
AGAP007032	7032	AmS046a	5'-CGAAAGCAGCAGAAGAATCG-3'	5'-TGCTGCATCGTTGTGCACG-3'	601	2007
AGAP007033	APL1C	AmS003a	5'-CTTCTGAATAGTGTGCGCGTAA-3'	5'-TGAGACAAACTTTGGAGGTCAG-3'	371	2007
AGAP007034	LRR(7034)	AmS047b	5'-CACAGATGCTCCAGCTTCG-3'	5'-CGTACTTGGTGGACCAACG-3'	738	2007
AGAP007035	APL1B	AmS036a	5'-AGATGGGTCTGTGTTTGCTG-3'	5'-CGCACAACCATTTGATGTGGG-3'	833	2007-2008
AGAP007036	APL1A	AmS037d	5'-TGTGATTTAYMCAACTCATGC-3'	5'-TCAAAGTGCTCGATYTGTCG-3'	802	2007
AGAP007037	LRR(7037)	AmS038a	5'-CGTTGTCCAGTATCCACAG-3'	5'-CAACAACACGATCAAGCAGC-3'	580	2007
AGAP007041	FBN32	AmS004b	5'-GTACGATGGTACGGTCGATTTC-3'	5'-GGTAGGAATGCTTTCCGATTAG-3'	486	2007-2008

Table S2: Continued

Identifier	Gene	PCR fragment	Forward oligo	Reverse oligo	Fragment Length	Year Sampled
AGAP007048	LRR(7048)	AmS005b	5'-TTTTTAAGCCTAGCCCGTCTG-3'	5'-CAGCTCGGTAAGCCGATTG-3'	491	2007-2008
AGAP007058	DLL	AmS007b	5'-GTACGTAGCCACCCATCTG-3'	5'-GTTAAGATCTGGTTTCAAAATCG-3'	641	2007-2008
AGAP007059	LRR(7059)	AmS051b	5'-ACCAGGCGCTAGTTCTTTGA-3'	5'-TACCGGCAACGGTCTTTAAC-3'	641	2007-2008
AGAP007060	LRR(7060)	AmS006b	5'-AGTAGCAGGCTCGTGAGTGAG-3'	5'-GAAGCACTTCCACTGGTGCT-3'	658	2007-2008
AGAP007061	LRR(7061)	AmS045b	5'-AGGAAAGATCAAGCAGCTCG-3'	5'-CTGGCGATCGTCAACAACG-3'	532	2007
H603flank	intergenic region	AmS050a	5'-CAAGGCAGCTTCTTCGTTCT-3'	5'-GTTCACGAGTTTGGTCTTGC-3'	522	2007-2008
AGAP001826	APOII/I	AmS056a	5'-CCGTTGACGTGGTACTTGG-3'	5'-ATGTGGCTGCCGATTTCTAC-3'	566	2007
AGAP002593	APOD	AmS057c	5'-GGTACGATCAACACTTCGAG-3'	5'-TGATGCGCATATCCTGTCG-3'	490	2007
AGAP010815	TEP1	AmS054c	5'-CGTATTTGGACGTCCGACG-3'	5'-CCATGCAATCAATGAGAACG-3'	579	2007
AGAP012352	AgMDL1	AmS055b	5'-CAGCAGGATTCACTGTTCTC-3'	5'-ATCCATGAGGTTTCGATCTC-3'	486	2007
AGAP001081	WASP	AmS058b	5'-TTCGTCCTCGGTAGCAAAG-3'	5'-TGGTGCAGCTGTACACGAC-3'	440	2007

Table S3 Sequenced fragment physical genomic locations

Identifier	Gene Name	Chromosome	Chromosomal Location[a]	Inside PRI?	Chromosomal Strand
AGAP005681	GPRNNA21	2L	18693376	Yes	-
AGAP005693	APL2	2L	18785249	Yes	+
AGAP005716	SCRB16	2L	19542880	Yes	+
AGAP005728	5728	2L	19769635	Yes	-
AGAP005762	5762	2L	20339460	Yes	-
AGAP006102	PRS1	2L	26685850	No	+
AGAP006348	LRIM1	2L	30329656	No	-
AGAP006421	IRSP1	2L	31693742	No	+
AGAP006974	TOLL9	2L	40434581	Yes	-
AGAP007030	LRR(7030)	2L	41057548	Yes	-
AGAP007032	7032	2L	41245076	Yes	+
AGAP007033	APL1C	2L	41257877	Yes	-
AGAP007034	LRR(7034)	2L	41262272	Yes	-
AGAP007035	APL1B	2L	41266619	Yes	-
AGAP007036	APL1A	2L	41271509	Yes	-
AGAP007037	LRR(7037)	2L	41274607	Yes	-
AGAP007041	FBN32	2L	41381834	Yes	+
AGAP007048	LRR(7048)	2L	41648960	Yes	+
AGAP007058	Distalless	2L	42005592	Yes	-
AGAP007059	LRR(7059)	2L	42005592	Yes	+
AGAP007060	LRR(7060)	2L	42062331	Yes	-
AGAP007061	LRR(7061)	2L	42067616	Yes	-
H603flank	intergenic region	2L	42071847	Yes	-
AGAP001826	APOII/I	2R	11201968	No	-
AGAP002593	APOD	2R	40909880	No	-
AGAP010815	TEP1	3L	11117128	No	-
AGAP012352	AgMDL1	3L	23677600	No	+
AGAP001081	WASP	X	23372448	No	-

[a] Gene starting positions on chromosome 2 given according to locations in *A. gambiae* PEST genome sequence, which corresponds to the inverted 2La+ form of the 2La inversion.

Table S4 Population genetic summary statistics for all genes in each population.

Locus	Identifier	n[a]	S_{syn}[b]	*D*[c]	*H*[d]	*EW*[e]	*HEW*[f]
M form							
Toll9	AGAP006974	64	79	-0.6612	-0.3681	0.019	1
LRR(7030)	AGAP007030	64	34	-0.7755	-0.8827	0.062	0.0921
APL1C	AGAP007033	64	21	-0.546	-1.1648	0.2739	0.084
FBN32	AGAP007041	100	33	-1.0852	-0.7677	0.0864	0.0992
LRR(7048)	AGAP007048	100	68	-2.0115	-0.1663	0.046	0.1441
LRR(7060)	AGAP007060	100	42	-1.6659	0.084	0.0842	0.2152
DLL	AGAP007058	100	87	-2.0234	-0.6157	0.0208	0.1036
GPRNNA21	AGAP005681	64	16	-0.7143	0.1597	0.1201	0.2411
APL2	AGAP005693	64	27	-1.0359	0.6826	0.0459	0.7155
SCRB16	AGAP005716	64	47	-0.3479	-0.3037	0.0347	0.1326
APL1B	AGAP007035	100	88	-0.5362	-1.7003	0.0898	0.0294
APL1A	AGAP007036	62	110	-1.15	-2.5176	0.3002	0.014
LRR(7037)	AGAP007037	64	13	-1.682	-0.2348	0.5239	0.1441
LRR(7061)	AGAP007061	64	37	-0.8415	0.1359	0.0605	0.2283
7032	AGAP007032	64	73	-1.4061	0.5115	0.0181	1
LRR(7034)	AGAP007034	62	20	0.174	0.7383	0.1623	0.779
5758	AGAP005758	64	29	-1.5625	-0.1749	0.0815	0.1441
5762	AGAP005762	64	73	-1.6686	-0.2999	0.02	0.1326
H603 flanking	AGAP007058	100	54	-1.866	-0.4372	0.023	0.1326
LRR(7059)	AGAP007059	100	50	-1.8131	-1.6246	0.148	0.0302
PRS1	AGAP006102	64	16	-1.6028	-0.1924	0.1709	0.1441
IRSP1	AGAP006421	64	55	-1.4194	-2.4365	0.0557	0.014
TEP1	AGAP010815	64	10	-2.1817	-2.0996	0.7979	0.0233
AgMDL2	AGAP012352	64	66	-1.7729	0.0863	0.0249	0.2667
APOII-I	AGAP001826	64	23	-1.5496	-0.2477	0.1338	0.1441
APOD	AGAP002593	64	37	-0.8402	-0.9231	0.0454	0.4199
WASP	AGAP001081	64	42	-1.6375	-0.3393	0.0322	0.1326
LRIM1	AGAP006348	64	25	-0.1096	-2.4951	0.1221	0.0336
S form							
Toll9	AGAP006974	100	81	-0.6536	-0.4918	0.0118	0.1352
LRR(7030)	AGAP007030	100	59	-1.1477	-1.1961	0.0378	0.091
APL1C	AGAP007033	100	72	-1.3893	-0.885	0.126	0.0928
FBN32	AGAP007041	100	47	-1.7597	-0.7475	0.0454	0.1117
LRR(7048)	AGAP007048	100	79	-2.2608	-0.1704	0.0364	0.1473

LRR(7060)	AGAP007060	100	50	-2.0471	-1.4173	0.0684	0.091
DLL	AGAP007058	100	96	-1.9502	-1.2775	0.0146	0.091
GPRNNA21	AGAP005681	100	28	-1.7265	-0.4675	0.1156	0.1473
APL2	AGAP005693	100	43	-1.7926	0.4499	0.0434	0.3721
SCRB16	AGAP005716	100	58	-0.7949	-0.4554	0.026	0.1352
APL1B	AGAP007035	100	120	-0.7912	-1.5837	0.0172	0.091
APL1A	AGAP007036	100	147	-0.061	-1.4618	0.0158	0.1352
LRR(7037)	AGAP007037	100	49	-2.0094	-0.0114	0.0688	0.1535
LRR(7061)	AGAP007061	100	33	-0.9212	-0.1171	0.0894	0.1473
7032	AGAP007032	100	83	-1.7822	0.2356	0.0154	0.2135
LRR(7034)	AGAP007034	100	42	-1.9241	-0.099	0.0598	0.1473
5758	AGAP005758	100	37	-1.7718	-0.1192	0.0642	0.1473
5762	AGAP005762	100	100	-1.564	0.0407	0.0122	0.1535
H603 flanking	AGAP007058	100	83	-2.2543	-0.1461	0.0202	0.1473
LRR(7059)	AGAP007059	100	51	-0.986	-0.2439	0.042	0.1473
PRS1	AGAP006102	100	17	-1.4331	-0.2205	0.1842	0.1473
IRSP1	AGAP006421	100	61	-0.8321	-0.9617	0.017	0.0928
TEP1	AGAP010815	100	15	0.317	0.565	0.2096	0.5221
AgMDL2	AGAP012352	100	68	-1.4512	0.4507	0.0198	0.3682
APOII-I	AGAP001826	100	44	-2.0695	0.1091	0.0598	0.1926
APOD	AGAP002593	100	53	-1.819	-0.9022	0.065	0.0928
WASP	AGAP001081	100	77	-2.2284	-0.1177	0.0432	0.1473
LRIM1	AGAP006348	100	47	-0.5435	-0.0234	0.0228	0.1535

GOUNDRY							
$2La^{+}/2La^{+}$							
Toll9	AGAP006974	100	50	-0.1716	-3.0625	0.0868	0.0224
LRR(7030)	AGAP007030	100	23	-0.1144	0.5018	0.059	0.5892
APL1C	AGAP007033	100	22	-0.1767	-1.6356	0.2556	0.0999
FBN32	AGAP007041	100	23	0.2029	0.3183	0.2676	0.425
LRR(7048)	AGAP007048	100	14	1.5159	-0.1189	0.1616	0.3041
LRR(7060)	AGAP007060	100	10	0.0003	-0.5047	0.3704	0.425
DLL	AGAP007058	100	16	0.9977	-0.2242	0.211	0.3027
GPRNNA21	AGAP005681	100	11	-0.4812	0.0792	0.365	0.3683
APL2	AGAP005693	100	13	-1.0815	-0.5745	0.3712	0.2448
SCRB16	AGAP005716	100	21	1.0104	-1.0902	0.1456	0.2402
APL1B	AGAP007035	100	70	0.7992	-2.3727	0.336	0.0999
APL1A	AGAP007036	100	63	2.4401	-1.8712	0.1678	0.7745
LRR(7037)	AGAP007037	100	8	0.3655	0.6926	0.2866	0.7745

LRR(7061)	AGAP007061	100	8	2.4058	0.072	0.418	0.425
7032	AGAP007032	100	19	1.1648	-3.0339	0.328	0.2402
LRR(7034)	AGAP007034	100	6	0.8999	0.4665	0.4092	0.7579
5758	AGAP005758	100	2	2.3379	0.5497	0.4616	0.7745
5762	AGAP005762	100	20	1.0203	-0.4835	0.2026	0.2448
H603 flanking	AGAP007058	100	16	-1.469	0.3172	0.3162	0.425
LRR(7059)	AGAP007059	100	9	1.6054	-0.0451	0.3622	0.425
PRS1	AGAP006102	100	14	-1.6311	0.2602	0.3306	0.499
IRSP1	AGAP006421	100	10	-0.5248	-1.9277	0.1946	0.425
TEP1	AGAP010815	100	11	0.1881	0.0061	0.3224	0.7133
AgMDL2	AGAP012352	100	48	-0.423	0.6236	0.1192	0.7126
APOII-I	AGAP001826	100	8	1.1057	0.6108	0.2666	0.7186
APOD	AGAP002593	100	38	-0.57	0.1383	0.0688	0.4118
WASP	AGAP001081	100	13	-0.8877	0.1567	0.392	0.4439
LRIM1	AGAP006348	100	22	0.8458	-0.2961	0.2756	0.2863
GOUNDRY							
$2La^a/2La^a$							
Toll9	AGAP006974	40	59	0.7813	-0.4603	0.0688	0.1255
LRR(7030)	AGAP007030	34	22	-1.3252	-1.5011	0.1211	0.0482
APL1C	AGAP007033	40	29	-0.1794	-0.6664	0.105	0.16
FBN32	AGAP007041	100	15	-1.2392	-1.6624	0.508	0.0482
LRR(7048)	AGAP007048	100	73	-1.6192	0.4025	0.1242	0.4164
LRR(7060)	AGAP007060	100	38	-2.0531	-2.2083	0.3992	0.028
DLL	AGAP007058	100	72	-2.3283	-2.5965	0.2512	0.0252
GPRNNA21	AGAP005681	40	9	-0.0237	-0.8588	0.3275	0.2329
APL2	AGAP005693	40	17	-1.4316	-0.7944	0.345	0.1101
SCRB16	AGAP005716	40	28	0.3575	-0.8282	0.1475	0.1033
APL1B	AGAP007035	100	70	0.2394	-2.4022	0.2408	0.0252
APL1A	AGAP007036	38	78	2.4679	-0.2821	0.2853	0.5291
LRR(7037)	AGAP007037	38	5	0.0936	-0.2734	0.4765	0.16
LRR(7061)	AGAP007061	40	19	-0.8833	-1.7333	0.2463	0.4164
7032	AGAP007032	40	36	-0.3302	0.3314	0.1038	0.3951
LRR(7034)	AGAP007034	40	10	1.2186	-1.045	0.4313	0.5291
5758	AGAP005758	38	9	1.173	0.9511	0.2175	0.931
5762	AGAP005762	38	25	-0.1673	0.4451	0.1177	0.4444
H603 flanking	AGAP007058	100	24	-1.312	-3.2452	0.1474	0.0994
LRR(7059)	AGAP007059	100	44	-0.924	-0.8104	0.1418	0.1033
PRS1	AGAP006102	40	8	0.5677	-1.2126	0.2888	0.1255

IRSP1	AGAP006421	40	34	-1.2448	-1.8962	0.22	0.1255
TEP1	AGAP010815	40	11	0.2615	0.3394	0.345	0.5291
AgMDL2	AGAP012352	40	44	-0.7708	0.7287	0.185	0.6782
APOII-I	AGAP001826	40	8	0.6999	0.5657	0.275	0.5291
APOD	AGAP002593	40	38	-1.0434	0.0154	0.08	0.2317
WASP	AGAP001081	38	16	-1.3667	0.3246	0.3213	0.4164
LRIM1	AGAP006348	38	17	2.1208	0.2522	0.3269	0.3668

a- Number of chromosomes in the sample. Loci with n=100 had more than 100 in the original sample, but were down-sampled to 100 for this analysis.

b- Number of synonymous segregating sites.

c- Tajima's *D* calculated using only synonymous sites.

d- Normalized Fay and Wu's *H* calculated using only synonymous sites.

e- Ewens-Watterson's haplotype homozygosity statistic calculated using only synonymous sites.

f- *HEW* *p*-value with Benjamini and Hochberg correction for multiple tests.

Statistical significance of *HEW* was evaluated by comparison to 10^5 neutral coalescent simulations of each sample (see Methods).

Table S5 Results from *post-hoc* evaluation of haploype reconstruction for genes with significant *HEW* result.

Gene	Confidence Rank[a]	Mean Probability (<1)[b]	Proportion Imputed Rank[c]	Proportion Phased Rank[d]
M form				
LRIM1	50	0.82	55	26
TEP1	20	0.76	90	100
APL1A	86	0.74	33	12
APL1B	76	0.81	36	19
LRR (7059)	40	0.78	70	72
IRSP1	24	0.73	35	32
GOUNDRY				
$2La^a/2La^a$				
DLL	53	0.73	27	70
APL1B	94	0.86	39	7
LRR (7030)	2	0.78	24	58
LRR (7060)	64	0.76	37	83
FBN32	74	0.77	62	88
GOUNDRY				
$2La^+/2La^+$				
TOLL9	63	0.78	29	24

Haplotype reconstruction and imputation was conducted for each gene in each population separately. For GOUNDRY, genes inside the 2La inversion were treated separately according to 2La homokaryotype, while genes outside of the inversion were treated as one group. In total, this resulted in 102 runs of the program PHASE (Stephens, Smith, and Donnelly 2001). When a heterozygous site is phased, it is given a statistical confidence probability ranging from 0.5 to 1, where 0.5 is low confidence or complete ambiguity and 1 being the highest level of confidence.

[a] Genes ranked based on the proportion of phased sites that were given a confidence probability less than 1, with 1 indicating the gene with the most

[b] Mean probability calculated for each gene for sites with probabilities less than 1.

[c] Genes ranked based on the proportion of sequenced sites that were imputed, with 1 representing gene with the most imputed sites.

[d] Genes ranked based on the proportion of sequenced sites that were phased, with 1 representing the gene with the most statistically phased sites.
