# Supplementary material for: Evidence for Population-Specific Positive Selection on Immune Genes of Anopheles gambiae
Source: G3 (Bethesda). 2012 Dec 1;2(12):1505–19. doi: 10.1534/g3.112.004473 (PMC3516473; doi:10.1534/g3.112.004473)
Supplement: Supporting Information [file supp_2.12.1505_TableS2.pdf]

Table S2 Fragment and PCR Oligo information.

Identifier	Gene	PCR fragment	Forward oligo	Reverse oligo	Fragment Length	Year Sampled
AGAP005681	GPRNNA21	AmS010b	5'-GCATCATCATCGGTCACCG-3'	5'-CTGAGTCACCTGCAAACCG-3'	528	2007
AGAP005693	APL2	AmS011b	5'-CTATCCACCGTCCAGTTTG-3'	5'-GGTTCGGTGGAATTCTAACC-3'	561	2007
AGAP005716	SCRB16	AmS013a	5'-TGCCGAAGATGAAACGTACG-3'	5'-CGTGCTAAAGATTGTCATCCG-3'	534	2007
AGAP005728	5728	AmS048b	5'-GAATTGCGCAAACAGTCCAG-3'	5'-CACGTTCGATATCTCGCTGA-3'	650	2007
AGAP005762	5762	AmS049b	5'-ATCGATGTCCTCGGCACTAC-3'	5'-GATGGTCAAAGCCAACGAAC-3'	525	2007
AGAP006102	PRS1	AmS052b	5'-CGAAAGTGATTCCGGACAAG-3'	5'-TTATCGCTCGCACAGCAC-3'	304	2007
AGAP006348	LRIM1	AmS059e	5'-CCTCGTACCGCTTGACGAT-3'	5'-GTGACCTGGATCAGTCTGC-3'	576	2007
AGAP006421	IRSP1	AmS053a	5'-ATGGCCATCTGGATAGCTTG-3'	5'-GATATTCGCTCCACCAGCTC-3'	563	2007
AGAP006974	TOLL9	AmS001b	5'-GCATCTCGAACTGACACCAG-3'	5'-TTCGGATATTCCGGAGGAG-3'	536	2007
AGAP007030	LRR(7030)	AmS002a	5'-AGAAACAACAGTCGCAAGCTC-3'	5'-ACATGCTGTGCACCATAAAGAA-3'	501	2007
AGAP007032	7032	AmS046a	5'-CGAAAGCAGCAGAAGAATCG-3'	5'-TGCTGCATCGTTGTGCACG-3'	601	2007
AGAP007033	APL1C	AmS003a	5'-CTTCTGAATAGTGTGCGCGTAA-3'	5'-TGAGACAAACTTTGGAGGTCAG-3'	371	2007
AGAP007034	LRR(7034)	AmS047b	5'-CACAGATGCTCCAGCTTCG-3'	5'-CGTACTTGGTGGACCAACG-3'	738	2007
AGAP007035	APL1B	AmS036a	5'-AGATGGGTCTGTGTTTGCTG-3'	5'-CGCACAACCATTTGATGTGGG-3'	833	2007-2008
AGAP007036	APL1A	AmS037d	5'-TGTGATTTAYMCAACTCATGC-3'	5'-TCAAAGTGCTCGATYTGTCG-3'	802	2007
AGAP007037	LRR(7037)	AmS038a	5'-CGTTGTCCAGTATCCACAG-3'	5'-CAACAACACGATCAAGCAGC-3'	580	2007
AGAP007041	FBN32	AmS004b	5'-GTACGATGGTACGGTCGATTTC-3'	5'-GGTAGGAATGCTTTCCGATTAG-3'	486	2007-2008

Table S2: Continued

Identifier	Gene	PCR fragment	Forward oligo	Reverse oligo	Fragment Length	Year Sampled
AGAP007048	LRR(7048)	AmS005b	5'-TTTTTAAGCCTAGCCCGTCTG-3'	5'-CAGCTCGGTAAGCCGATTG-3'	491	2007-2008
AGAP007058	DLL	AmS007b	5'-GTACGTAGCCACCCATCTG-3'	5'-GTTAAGATCTGGTTTCAAAATCG-3'	641	2007-2008
AGAP007059	LRR(7059)	AmS051b	5'-ACCAGGCGCTAGTTCTTTGA-3'	5'-TACCGGCAACGGTCTTTAAC-3'	641	2007-2008
AGAP007060	LRR(7060)	AmS006b	5'-AGTAGCAGGCTCGTGAGTGAG-3'	5'-GAAGCACTTCCACTGGTGCT-3'	658	2007-2008
AGAP007061	LRR(7061)	AmS045b	5'-AGGAAAGATCAAGCAGCTCG-3'	5'-CTGGCGATCGTCAACAACG-3'	532	2007
H603flank	intergenic region	AmS050a	5'-CAAGGCAGCTTCTTCGTTCT-3'	5'-GTTCACGAGTTTGGTCTTGC-3'	522	2007-2008
AGAP001826	APOII/I	AmS056a	5'-CCGTTGACGTGGTACTTGG-3'	5'-ATGTGGCTGCCGATTTCTAC-3'	566	2007
AGAP002593	APOD	AmS057c	5'-GGTACGATCAACACTTCGAG-3'	5'-TGATGCGCATATCCTGTCG-3'	490	2007
AGAP010815	TEP1	AmS054c	5'-CGTATTTGGACGTCCGACG-3'	5'-CCATGCAATCAATGAGAACG-3'	579	2007
AGAP012352	AgMDL1	AmS055b	5'-CAGCAGGATTCACTGTTCTC-3'	5'-ATCCATGAGGTTTCGATCTC-3'	486	2007
AGAP001081	WASP	AmS058b	5'-TTCGTCCTCGGTAGCAAAG-3'	5'-TGGTGCAGCTGTACACGAC-3'	440	2007
