# Supplementary material for: Evidence for Population-Specific Positive Selection on Immune Genes of Anopheles gambiae
Source: G3 (Bethesda). 2012 Dec 1;2(12):1505–19. doi: 10.1534/g3.112.004473 (PMC3516473; doi:10.1534/g3.112.004473)
Supplement: Supporting Information [file supp_2.12.1505_TableS3.pdf]

Table S3 Sequenced fragment physical genomic locations

Identifier	Gene Name	Chromosome	Chromosomal Location[a]	Inside PRI?	Chromosomal Strand
AGAP005681	GPRNNA21	2L	18693376	Yes	-
AGAP005693	APL2	2L	18785249	Yes	+
AGAP005716	SCRB16	2L	19542880	Yes	+
AGAP005728	5728	2L	19769635	Yes	-
AGAP005762	5762	2L	20339460	Yes	-
AGAP006102	PRS1	2L	26685850	No	+
AGAP006348	LRIM1	2L	30329656	No	-
AGAP006421	IRSP1	2L	31693742	No	+
AGAP006974	TOLL9	2L	40434581	Yes	-
AGAP007030	LRR(7030)	2L	41057548	Yes	-
AGAP007032	7032	2L	41245076	Yes	+
AGAP007033	APL1C	2L	41257877	Yes	-
AGAP007034	LRR(7034)	2L	41262272	Yes	-
AGAP007035	APL1B	2L	41266619	Yes	-
AGAP007036	APL1A	2L	41271509	Yes	-
AGAP007037	LRR(7037)	2L	41274607	Yes	-
AGAP007041	FBN32	2L	41381834	Yes	+
AGAP007048	LRR(7048)	2L	41648960	Yes	+
AGAP007058	Distalless	2L	42005592	Yes	-
AGAP007059	LRR(7059)	2L	42005592	Yes	+
AGAP007060	LRR(7060)	2L	42062331	Yes	-
AGAP007061	LRR(7061)	2L	42067616	Yes	-
H603flank	intergenic region	2L	42071847	Yes	-
AGAP001826	APOII/I	2R	11201968	No	-
AGAP002593	APOD	2R	40909880	No	-
AGAP010815	TEP1	3L	11117128	No	-
AGAP012352	AgMDL1	3L	23677600	No	+
AGAP001081	WASP	X	23372448	No	-

[a] Gene starting positions on chromosome 2 given according to locations in *A. gambiae* PEST genome sequence, which corresponds to the inverted 2La+ form of the 2La inversion.
