# Supplementary material for: Evidence for Population-Specific Positive Selection on Immune Genes of Anopheles gambiae
Source: G3 (Bethesda). 2012 Dec 1;2(12):1505–19. doi: 10.1534/g3.112.004473 (PMC3516473; doi:10.1534/g3.112.004473)
Supplement: Supporting Information [file supp_2.12.1505_TableS4.pdf]

Table S4 Population genetic summary statistics for all genes in each population.

Locus	Identifier	n[a]	S_{syn}[b]	*D*[c]	*H*[d]	*EW*[e]	*HEW*[f]
M form							
Toll9	AGAP006974	64	79	-0.6612	-0.3681	0.019	1
LRR(7030)	AGAP007030	64	34	-0.7755	-0.8827	0.062	0.0921
APL1C	AGAP007033	64	21	-0.546	-1.1648	0.2739	0.084
FBN32	AGAP007041	100	33	-1.0852	-0.7677	0.0864	0.0992
LRR(7048)	AGAP007048	100	68	-2.0115	-0.1663	0.046	0.1441
LRR(7060)	AGAP007060	100	42	-1.6659	0.084	0.0842	0.2152
DLL	AGAP007058	100	87	-2.0234	-0.6157	0.0208	0.1036
GPRNNA21	AGAP005681	64	16	-0.7143	0.1597	0.1201	0.2411
APL2	AGAP005693	64	27	-1.0359	0.6826	0.0459	0.7155
SCRB16	AGAP005716	64	47	-0.3479	-0.3037	0.0347	0.1326
APL1B	AGAP007035	100	88	-0.5362	-1.7003	0.0898	0.0294
APL1A	AGAP007036	62	110	-1.15	-2.5176	0.3002	0.014
LRR(7037)	AGAP007037	64	13	-1.682	-0.2348	0.5239	0.1441
LRR(7061)	AGAP007061	64	37	-0.8415	0.1359	0.0605	0.2283
7032	AGAP007032	64	73	-1.4061	0.5115	0.0181	1
LRR(7034)	AGAP007034	62	20	0.174	0.7383	0.1623	0.779
5758	AGAP005758	64	29	-1.5625	-0.1749	0.0815	0.1441
5762	AGAP005762	64	73	-1.6686	-0.2999	0.02	0.1326
H603 flanking	AGAP007058	100	54	-1.866	-0.4372	0.023	0.1326
LRR(7059)	AGAP007059	100	50	-1.8131	-1.6246	0.148	0.0302
PRS1	AGAP006102	64	16	-1.6028	-0.1924	0.1709	0.1441
IRSP1	AGAP006421	64	55	-1.4194	-2.4365	0.0557	0.014
TEP1	AGAP010815	64	10	-2.1817	-2.0996	0.7979	0.0233
AgMDL2	AGAP012352	64	66	-1.7729	0.0863	0.0249	0.2667
APOII-I	AGAP001826	64	23	-1.5496	-0.2477	0.1338	0.1441
APOD	AGAP002593	64	37	-0.8402	-0.9231	0.0454	0.4199
WASP	AGAP001081	64	42	-1.6375	-0.3393	0.0322	0.1326
LRIM1	AGAP006348	64	25	-0.1096	-2.4951	0.1221	0.0336
S form							
Toll9	AGAP006974	100	81	-0.6536	-0.4918	0.0118	0.1352
LRR(7030)	AGAP007030	100	59	-1.1477	-1.1961	0.0378	0.091
APL1C	AGAP007033	100	72	-1.3893	-0.885	0.126	0.0928
FBN32	AGAP007041	100	47	-1.7597	-0.7475	0.0454	0.1117
LRR(7048)	AGAP007048	100	79	-2.2608	-0.1704	0.0364	0.1473

LRR(7060)	AGAP007060	100	50	-2.0471	-1.4173	0.0684	0.091
DLL	AGAP007058	100	96	-1.9502	-1.2775	0.0146	0.091
GPRNNA21	AGAP005681	100	28	-1.7265	-0.4675	0.1156	0.1473
APL2	AGAP005693	100	43	-1.7926	0.4499	0.0434	0.3721
SCRB16	AGAP005716	100	58	-0.7949	-0.4554	0.026	0.1352
APL1B	AGAP007035	100	120	-0.7912	-1.5837	0.0172	0.091
APL1A	AGAP007036	100	147	-0.061	-1.4618	0.0158	0.1352
LRR(7037)	AGAP007037	100	49	-2.0094	-0.0114	0.0688	0.1535
LRR(7061)	AGAP007061	100	33	-0.9212	-0.1171	0.0894	0.1473
7032	AGAP007032	100	83	-1.7822	0.2356	0.0154	0.2135
LRR(7034)	AGAP007034	100	42	-1.9241	-0.099	0.0598	0.1473
5758	AGAP005758	100	37	-1.7718	-0.1192	0.0642	0.1473
5762	AGAP005762	100	100	-1.564	0.0407	0.0122	0.1535
H603 flanking	AGAP007058	100	83	-2.2543	-0.1461	0.0202	0.1473
LRR(7059)	AGAP007059	100	51	-0.986	-0.2439	0.042	0.1473
PRS1	AGAP006102	100	17	-1.4331	-0.2205	0.1842	0.1473
IRSP1	AGAP006421	100	61	-0.8321	-0.9617	0.017	0.0928
TEP1	AGAP010815	100	15	0.317	0.565	0.2096	0.5221
AgMDL2	AGAP012352	100	68	-1.4512	0.4507	0.0198	0.3682
APOII-I	AGAP001826	100	44	-2.0695	0.1091	0.0598	0.1926
APOD	AGAP002593	100	53	-1.819	-0.9022	0.065	0.0928
WASP	AGAP001081	100	77	-2.2284	-0.1177	0.0432	0.1473
LRIM1	AGAP006348	100	47	-0.5435	-0.0234	0.0228	0.1535
GOUNDRY							
$2La^+/2La^+$							
Toll9	AGAP006974	100	50	-0.1716	-3.0625	0.0868	0.0224
LRR(7030)	AGAP007030	100	23	-0.1144	0.5018	0.059	0.5892
APL1C	AGAP007033	100	22	-0.1767	-1.6356	0.2556	0.0999
FBN32	AGAP007041	100	23	0.2029	0.3183	0.2676	0.425
LRR(7048)	AGAP007048	100	14	1.5159	-0.1189	0.1616	0.3041
LRR(7060)	AGAP007060	100	10	0.0003	-0.5047	0.3704	0.425
DLL	AGAP007058	100	16	0.9977	-0.2242	0.211	0.3027
GPRNNA21	AGAP005681	100	11	-0.4812	0.0792	0.365	0.3683
APL2	AGAP005693	100	13	-1.0815	-0.5745	0.3712	0.2448
SCRB16	AGAP005716	100	21	1.0104	-1.0902	0.1456	0.2402
APL1B	AGAP007035	100	70	0.7992	-2.3727	0.336	0.0999
APL1A	AGAP007036	100	63	2.4401	-1.8712	0.1678	0.7745
LRR(7037)	AGAP007037	100	8	0.3655	0.6926	0.2866	0.7745

LRR(7061)	AGAP007061	100	8	2.4058	0.072	0.418	0.425
7032	AGAP007032	100	19	1.1648	-3.0339	0.328	0.2402
LRR(7034)	AGAP007034	100	6	0.8999	0.4665	0.4092	0.7579
5758	AGAP005758	100	2	2.3379	0.5497	0.4616	0.7745
5762	AGAP005762	100	20	1.0203	-0.4835	0.2026	0.2448
H603 flanking	AGAP007058	100	16	-1.469	0.3172	0.3162	0.425
LRR(7059)	AGAP007059	100	9	1.6054	-0.0451	0.3622	0.425
PRS1	AGAP006102	100	14	-1.6311	0.2602	0.3306	0.499
IRSP1	AGAP006421	100	10	-0.5248	-1.9277	0.1946	0.425
TEP1	AGAP010815	100	11	0.1881	0.0061	0.3224	0.7133
AgMDL2	AGAP012352	100	48	-0.423	0.6236	0.1192	0.7126
APOII-I	AGAP001826	100	8	1.1057	0.6108	0.2666	0.7186
APOD	AGAP002593	100	38	-0.57	0.1383	0.0688	0.4118
WASP	AGAP001081	100	13	-0.8877	0.1567	0.392	0.4439
LRIM1	AGAP006348	100	22	0.8458	-0.2961	0.2756	0.2863
GOUNDRY							
$2La^a/2La^a$							
Toll9	AGAP006974	40	59	0.7813	-0.4603	0.0688	0.1255
LRR(7030)	AGAP007030	34	22	-1.3252	-1.5011	0.1211	0.0482
APL1C	AGAP007033	40	29	-0.1794	-0.6664	0.105	0.16
FBN32	AGAP007041	100	15	-1.2392	-1.6624	0.508	0.0482
LRR(7048)	AGAP007048	100	73	-1.6192	0.4025	0.1242	0.4164
LRR(7060)	AGAP007060	100	38	-2.0531	-2.2083	0.3992	0.028
DLL	AGAP007058	100	72	-2.3283	-2.5965	0.2512	0.0252
GPRNNA21	AGAP005681	40	9	-0.0237	-0.8588	0.3275	0.2329
APL2	AGAP005693	40	17	-1.4316	-0.7944	0.345	0.1101
SCRB16	AGAP005716	40	28	0.3575	-0.8282	0.1475	0.1033
APL1B	AGAP007035	100	70	0.2394	-2.4022	0.2408	0.0252
APL1A	AGAP007036	38	78	2.4679	-0.2821	0.2853	0.5291
LRR(7037)	AGAP007037	38	5	0.0936	-0.2734	0.4765	0.16
LRR(7061)	AGAP007061	40	19	-0.8833	-1.7333	0.2463	0.4164
7032	AGAP007032	40	36	-0.3302	0.3314	0.1038	0.3951
LRR(7034)	AGAP007034	40	10	1.2186	-1.045	0.4313	0.5291
5758	AGAP005758	38	9	1.173	0.9511	0.2175	0.931
5762	AGAP005762	38	25	-0.1673	0.4451	0.1177	0.4444
H603 flanking	AGAP007058	100	24	-1.312	-3.2452	0.1474	0.0994
LRR(7059)	AGAP007059	100	44	-0.924	-0.8104	0.1418	0.1033
PRS1	AGAP006102	40	8	0.5677	-1.2126	0.2888	0.1255

IRSP1	AGAP006421	40	34	-1.2448	-1.8962	0.22	0.1255
TEP1	AGAP010815	40	11	0.2615	0.3394	0.345	0.5291
AgMDL2	AGAP012352	40	44	-0.7708	0.7287	0.185	0.6782
APOII-I	AGAP001826	40	8	0.6999	0.5657	0.275	0.5291
APOD	AGAP002593	40	38	-1.0434	0.0154	0.08	0.2317
WASP	AGAP001081	38	16	-1.3667	0.3246	0.3213	0.4164
LRIM1	AGAP006348	38	17	2.1208	0.2522	0.3269	0.3668

a- Number of chromosomes in the sample. Loci with n=100 had more than 100 in the original sample, but were down-sampled to 100 for this analysis.

b- Number of synonymous segregating sites.

c- Tajima's *D* calculated using only synonymous sites.

d- Normalized Fay and Wu's *H* calculated using only synonymous sites.

e- Ewens-Watterson's haplotype homozygosity statistic calculated using only synonymous sites.

f- *HEW p*-value with Benjamini and Hochberg correction for multiple tests.

Statistical significance of *HEW* was evaluated by comparison to 10^5 neutral coalescent simulations of each sample (see Methods).
