# Supplementary material for: Evidence for Population-Specific Positive Selection on Immune Genes of Anopheles gambiae
Source: G3 (Bethesda). 2012 Dec 1;2(12):1505–19. doi: 10.1534/g3.112.004473 (PMC3516473; doi:10.1534/g3.112.004473)
Supplement: Supporting Information [file supp_2.12.1505_TableS5.pdf]

Table S5 Results from *post-hoc* evaluation of haploype reconstruction for genes with significant *HEW* result.

Gene	Confidence Rank[a]	Mean Probability (<1)[b]	Proportion Imputed Rank[c]	Proportion Phased Rank[d]
M form				
LRIM1	50	0.82	55	26
TEP1	20	0.76	90	100
APL1A	86	0.74	33	12
APL1B	76	0.81	36	19
LRR (7059)	40	0.78	70	72
IRSP1	24	0.73	35	32
GOUNDRY				
$2La^{a}/2La^{a}$				
DLL	53	0.73	27	70
APL1B	94	0.86	39	7
LRR (7030)	2	0.78	24	58
LRR (7060)	64	0.76	37	83
FBN32	74	0.77	62	88
GOUNDRY				
$2La^{+}/2La^{+}$				
TOLL9	63	0.78	29	24

Haplotype reconstruction and imputation was conducted for each gene in each population separately. For GOUNDRY, genes inside the 2La inversion were treated separately according to 2La homokaryotype, while genes outside of the inversion were treated as one group. In total, this resulted in 102 runs of the program PHASE (Stephens, Smith, and Donnelly 2001). When a heterozygous site is phased, it is given a statistical confidence probability ranging from 0.5 to 1, where 0.5 is low confidence or complete ambiguity and 1 being the highest level of confidence.

[a] Genes ranked based on the proportion of phased sites that were given a confidence probability less than 1, with 1 indicating the gene with the most

[b] Mean probability calculated for each gene for sites with probabilities less than 1.

[c] Genes ranked based on the proportion of sequenced sites that were imputed, with 1 representing gene with the most imputed sites.

[d] Genes ranked based on the proportion of sequenced sites that were phased, with 1 representing the gene with the most statistically phased sites.
